# The Probable Cell of Origin of NF1- and PDGF-Driven Glioblastomas

**DOI:** 10.1371/journal.pone.0024454

**Published:** 2011-09-09

**Authors:** Dolores Hambardzumyan, Yu-Kang Cheng, Hiroshi Haeno, Eric C. Holland, Franziska Michor

**Affiliations:** 1 Department of Regenerative Medicine, Cleveland Clinic, Cleveland, Ohio, United States of America; 2 Department of Biostatistics and Computational Biology, Dana-Farber Cancer Institute, and Department of Biostatistics, Harvard School of Public Health, Boston, Massachusetts, United States of America; 3 Cancer Biology and Genetics Program, Memorial Sloan-Kettering Cancer Center, New York, New York, United States of America; 4 Tri-Institutional Training Program in Computational Biology and Medicine, Weill Cornell Medical College, New York, New York, United States of America; 5 Department of Neurosurgery, Brain Tumor Center, Memorial Sloan Kettering Cancer Center, New York, New York, United States of America; 6 Department of Cancer Biology and Genetics, Memorial Sloan Kettering Cancer Center, New York, New York, United States of America; 7 Department of Neurology, Memorial Sloan Kettering Cancer Center, New York, New York, United States of America; 8 Department of Surgery, Memorial Sloan Kettering Cancer Center, New York, New York, United States of America; Genentech Inc., United States of America

## Abstract

Primary glioblastomas are subdivided into several molecular subtypes. There is an ongoing debate over the cell of origin for these tumor types where some suggest a progenitor while others argue for a stem cell origin. Even within the same molecular subgroup, and using lineage tracing in mouse models, different groups have reached different conclusions. We addressed this problem from a combined mathematical modeling and experimental standpoint. We designed a novel mathematical framework to identify the most likely cells of origin of two glioma subtypes. Our mathematical model of the unperturbed *in vivo* system predicts that if a genetic event contributing to tumor initiation imparts symmetric self-renewing cell division (such as PDGF overexpression), then the cell of origin is a transit amplifier. Otherwise, the initiating mutations arise in stem cells. The mathematical framework was validated with the RCAS/tv-a system of somatic gene transfer in mice. We demonstrated that PDGF-induced gliomas can be derived from GFAP-expressing cells of the subventricular zone or the cortex (reactive astrocytes), thus validating the predictions of our mathematical model. This interdisciplinary approach allowed us to determine the likelihood that individual cell types serve as the cells of origin of gliomas in an unperturbed system.

## Introduction

Glioblastomas (GBMs) are the most common primary brain tumors [1]. Over the years, our understanding of GBM biology has greatly improved, but the cell of origin for these tumors is still debated [2]. Some studies have demonstrated that neural stem cells (NSCs), located in the subventricular zone (SVZ), are a possible target for transformation [2]. NSCs in the SVZ and in the subgranular zone (SGZ) are capable of self-renewal and give rise to the three cell types in the central nervous system [3]. However, several studies suggested that extracellular signals can affect glial cell specification and may convert specified precursors into multipotential stem cells [4]. The cerebral cortex contains many cell types including astrocytes and oligodendroglial progenitor cells (OPCs) that have been reported to behave stem-like under certain culture or pathologic conditions [4], [5]. In fact, several of the major genetic alterations associated with gliomas confer some of the properties of stem cells [6].

Genomic and expression analyses of GBMs imply that they are not a single tumor type, but fall into several distinct subtypes; similarly, the cell of origin for these glioma types may well not be the same. Two studies have used correlative expression profiling to subdivide the tumors into groups based on similarities to known cell types named either proneural, proliferative, and mesenchymal [7], or proneural, neural, classical, and mesenchymal [8]. In addition, a combination of proteomic and genomic analyses have been used to subdivide these tumors into three subclasses based on signal transduction pathway activation and genetic alterations: the NF1, EGFR and PDGFR classes [9].

Mutations in the *NF1* gene have long been known to predispose to glioma formation, as these tumors are part of the tumor spectrum of the NF syndrome [10]. Recently, The Cancer Genome Atlas (TCGA) consortium showed that a surprisingly large number of sporadic GBMs have *NF1* mutations and define a subgroup of GBM [9], [11]. Genetic modeling of this GBM subtype in mice achieved by deleting *NF1*, *TP53* and/or *PTEN* in nestin-expressing cells (NSCs/PC) and GFAP-expressing cells (NSCs and white matter astrocytes) results in tumors histologically identical to human gliomas [12]. Moreover, stereotactic injection of *cre*-expressing adenovirus further demonstrated that these tumors could be initiated in cells of the SVZ (including NSCs and PCs) but not in more differentiated cells residing in the cortex [12]. In apparent contrast to these findings, recent usage of Mosaic Analysis with Double Markers (MADM) identified oligodendrocyte precursor cells (OPCs) to be the cell of origin in gliomas induced by concurrent mutation of *TP53* and *NF1* [13]. Loss of *NF1* was shown to transiently promote self-renewal [14], but acquisition of long-term self-renewal by *NF1* loss has not been reported.

A well-documented alteration in GBM is amplification and activating mutation (*EGFRVIII*) of *EGFR*. TCGA analysis of gliomas shows this mutation to be frequently associated with PTEN loss and to be the signature lesion of the EGFR subset of GBMs [9], [11]. Modeling of this subtype of GBM has been achieved in adult mice by adenoviral Cre delivery to the striatum to activate expression of *EGFR* (both *WT* and *VIII*) with simultaneous inactivation of *PTEN* and *INK4A/ARF* [15]. As adenoviral vectors are not cell type-specific, the cell of origin for these *cre*-induced tumors is not well-defined.

The third major signaling subset of gliomas is characterized by dysregulated PDGFR activity, which in some cases is due to amplification and rearrangement of the *PDGFR-α* gene locus, and in others to overexpression of the PDGF ligand [9]. The PDGFR subclass accounts for 25-30% of GBMs and overlaps with the proneural transcriptomal class of GBMs. The PDGF subset of gliomas has been modeled effectively using either MLV-based retroviral gene transfer or RCAS-mediated gene transfer in rodents [16], [17], [18]. These tumors can arise in neonatal or adult mice from either GFAP- or nestin-expressing cells, representing differentiated and self-renewing cells, respectively. Stereotactic injection of RCAS vector-mediated PDGF-induced gliomas from nestin-expressing cells shows similar incidence and latency when injected in the SVZ and the cortex of adult mice [18]. Although nestin is induced in the cortex by injury, the fact that nestin is also a stem cell/progenitor marker clouds the interpretation of these results. However, OPCs can also serve as the cells of origin for these gliomas. RCAS-PDGF injection into the cortex of *Ctv-a* (2′,3′-cyclic nucleotide 3′-phosphodiesterase (*Cnp*) *cnp-tv-a*) mice results in efficient glioma formation [19]. Recent studies of a mouse model of glioma driven by v-ErbB further validated that OPCs rather than NSCs serve as the cell of origin for murine oligodendroglioma [20].

The identity of the cell of origin for cancer has recently received much attention [21]. Different cells of origin could explain phenotypic differences between tumors associated with the same characteristic molecular abnormalities [22], [23], [24]. Furthermore, different cells of origin may require different combinations of oncogenic mutations that could affect the biology and/or therapeutic response of the tumors. It is therefore of significant interest to identify the cell of origin of human tumors. In this context, we define the cell of origin to be the first cell in which all genetic alterations necessary to initiate tumorigenesis are accumulated. This cell may be distinct from “tumor-propagating” cells, which maintain the tumor bulk [21].

In this paper, we have designed and analyzed a mathematical framework to identify the most likely cell of origin of glioblastoma. Given that previous mouse experiments have shown that PDGF overexpression may drive de-differentiation of glial cells [16], and recent mouse models have acknowledged the possibility of forcing a non-self-renewing cell to become the cell of origin [18], we sought to mathematically determine how likely such a non-stem cell origin may be. Thus, our model investigates a mechanism (cellular dedifferentiation induced by factors such as PDGF) by which such a cell of origin may occur and tests over a wide set of parameter regimes estimated from the glioma literature (see the Results section) whether and how often this mechanism is used. We considered two different subsets of gliomas: those that are driven by overexpression of PDGF, and those driven by loss of *NF1*. We found that if a genetic event contributing to tumor initiation imparts symmetric self-renewing cell division (such as PDGF overexpression), then the most likely cell of origin is a transit amplifier. If no such mutation emerges, then the cell of origin is a stem cell. These mathematical modeling experiments can be used to determine the likelihood and circumstances under which specific cell types initiate PDGF- and NF1-induced gliomagenesis. This approach is unlike mouse modeling experiments, which can only determine the ability of a certain cell type to initiate glioma development in response to overexpression of a gene, such as in the PDGF-driven case, or loss of function of a gene, as in the NF1-driven case, at a particular point in time. Mouse modeling does not ensure that the set of events that result in a particular cell of origin actually occurs *in vivo*. Thus, mouse modeling approaches cannot address the relative likelihood of different cell types to serve as the cell of origin in the unperturbed, non-engineered system evolving due to spontaneous mutation. The key advantage of mathematical modeling is that the evolution of human tumors can be recapitulated without having to impose the emergence of mutations at specific times, such as in the mouse model. This fact makes mathematical modeling uniquely able to address the identity of the cell of origin in naturally evolving tumors. However, mouse experiments are useful for validating a subset of predictions of the mathematical model – for instance, as outlined in the Results section, the mathematical framework predicts that gliomas driven by a genetic event which imparts symmetric self-renewing cell division (such as PDGF) should be able to be induced in all locations within the brain, while gliomas driven by genetic events which do not impart such characteristics can only arise from neural stem cells. To validate these predictions, we used the RCAS/tv-a system to target PDGF expression to GFAP-expressing cells of the SVZ, cortex and cerebellum. We found that PDGF expression in all three locations led to glioma formation. These results are in marked contrast to one set of experiments performed with the NF1-driven subclass of gliomas; stereotactic injection of cre recombinase to remove floxed *NF1* and *TP53* resulted in glioma formation only in the SVZ and not in the cortex or cerebellum [12]. This interdisciplinary approach of mathematical modeling and experimental validation provides a powerful new way of investigating the cell of origin of human tumors.

## Results

### Mathematical modeling of gliomagenesis identifies the most likely targets of transformation for different glioma subtypes

We designed a mathematical framework of the dynamics of proliferating cells in the brain, consisting of self-renewing (SR) cells and their transit-amplifying, non-self-renewing progeny (TA cells) (see Supporting Information S1 for details of the framework). We chose to begin with a detailed stochastic simulation in order to better capture the intricacies of the cell populations of the brain. Simpler analytic models may represent portions of the system dynamics [25]; however, they can only provide an approximate understanding of how the system functions in detail. To accurately capture the intricacies of the full system, an extended model must be designed that includes further parameters which may alter the conclusions of any simpler model [26].

We first modeled the population of SR cells residing in the SVZ. We considered this population to consist of multiple independent cell clusters or niches of 1 to 10 cells each [27] and denoted the number of SR cells in each cluster by *N*. We investigated the dynamics of one niche within the brain since the total probability of cancer initiation is given by the probability per niche times the number of niches, since the niches are independent. Hence, a consideration of all niches does not alter the most likely cell of origin of brain cancer. Within a niche, a single SR cell is randomly chosen to divide during each time step, following a stochastic process known as the Moran model [28]. According to this model, one daughter cell of the division replaces the divided cell; the other daughter cell replaces another cell in the population, which undergoes apoptosis. During each division, a mutation may occur in either one of the daughter cells.

**Figure 1:**
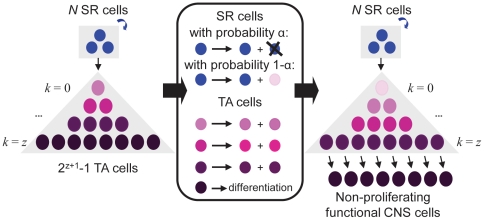
A mathematical model of the cell of origin of PDGF- and NF1-driven gliomas. Initially, there are *N* wild-type self-renewing (SR) cells (blue) and 2*^z^* ^+1^−1 wild-type transit-amplifying non-self-renewing (TA) cells (purple). At each time step, a SR cell is chosen to divide. With probability *α*, the SR cell divides symmetrically and one daughter cell replaces another randomly chosen SR cell. With probability 1−*α*, the SR cell divides asymmetrically and one daughter cell remains a SR cell while the other daughter cell becomes committed to the TA population (light pink). This new TA cell divides symmetrically *z* times to give rise to successively more differentiated cells (progressively darker shades of purple) before becoming terminally differentiated. This restriction of the stochastic process ensures that the total number of cells is constant over time, and mimics homeostatic conditions in the healthy brain. In the figure, the darkening purple gradations refer to successively more differentiated cells and serve to clarify a single time step of the stochastic process. We investigate the dynamics of only one cell cluster since the total probability of cancer initiation is given by the probability per cluster times the number of clusters; hence, a consideration of all clusters does not alter the identity of the most likely cell of origin of brain cancer.

SR cells do not produce only other SR cells. With probability *α*, the cell division in the current time step is symmetric and follows the traditional Moran process, while with probability 1−*α*, the SR division is asymmetric and results in one self-renewing and one non-self-renewing cell. In the latter case, the SR daughter cell replaces the divided cell, and the TA daughter cell begins a differentiation cascade. With each asymmetric SR cell division, all TA cells in the simulation undergo symmetric divisions, producing two daughter cells of successively more differentiated status (Fig. 1). Unmutated TA cells undergo *z* symmetric cell divisions before terminally differentiating; at this point, they are no longer considered in our mathematical model. Thus, in the wild-type system, there are *N* SR and 2*^z^* ^+1^−1 TA cells per niche.

During each SR and TA cell division, genetic alterations contributing to gliomagenesis may arise. We considered two subtypes of primary glioblastoma: the subtype driven by *NF1* loss and the subtype driven by PDGF overexpression. For NF1-driven cancers, we investigated bi-allelic loss of *NF1* and a dominant negative mutation of *TP53* as the necessary driver mutations that must be accumulated in a single cell to initiate tumorigenesis. For PDGF-driven cancers, the necessary driving alterations are those leading to PDGF overexpression and bi-allelic loss of *INK4A/ARF*. We did not include the accumulation of passenger mutations in this model since those alterations, by definition, do not influence the systems dynamics. The rates at which alterations leading to loss of *NF1*, *INK4A/ARF*, and *TP53*, and overexpression of PDGF arise per allele per cell division were denoted by *μ_NF1_*, *μ_ARF_*, *μ_TP53_*, and *μ_PDGF_* respectively. For both glioma subtypes, we assumed that a single cell that accumulates all required mutations and either retains or acquires the ability to self-renew is necessary and sufficient to initiate cancer. The cell of origin was then defined as the cell in which the last one of the necessary alterations emerges; this choice was made since the clone carrying the first mutation may often go extinct or not accumulate the other alterations necessary for tumor initiation.

**Figure 2:**
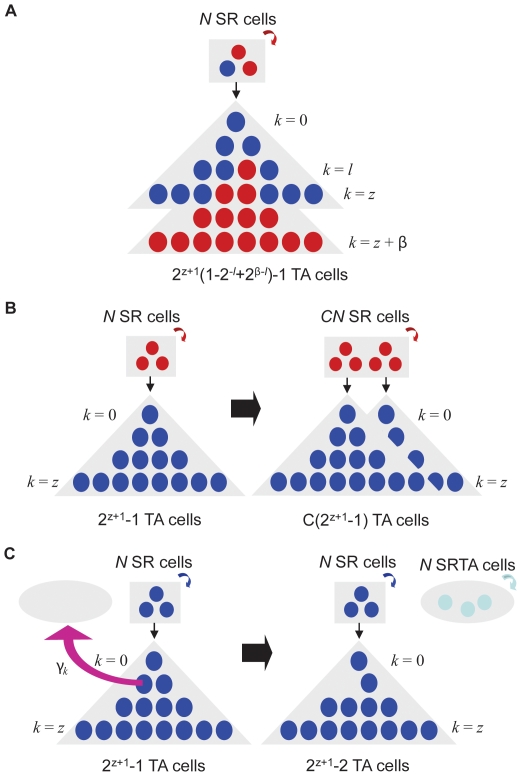
The effects of genetic alterations contributing to gliomagenesis. **A**) The acquisition of inactivating mutations in both alleles of *INK4A/ARF*, both alleles of *NF1*, and a dominant negative mutation in *TP53* all result in an increased relative fitness (i.e. growth rate) of SR and SRTA cells (red) as compared to wild type cells (blue) and an increased number of divisions TA cells can undergo before terminally differentiating. *INK4A/ARF^−/−^* and *TP53* dominant negative cells have relative fitness values of *R_ARF_* and *R_TP53_*, respectively, while *NF1^−/−^* cells have differing relative fitness values depending on the other mutations they harbor. If inactivating mutations in *TP53* or *INK4A/ARF* are present in the same cell, then *NF1^−/−^* cells have relative fitness *R_NF1,mut_*; if those mutations are not present, they have relative fitness *R_NF1,wt_*. Each mutation provides an additional *β_ARF_*, *β_NF1_*, or *β_TP53_* divisions that TA cells can undergo; hence *INK4A/ARF^−/−^*cells divide *z*+*β_ARF_* times instead of the usual *z* times. However, the additional divisions due to *NF1* loss only occur if either the *TP53* or the *INK4A/ARF* mutation has already been accumulated. **B**) If a cell overexpressing PDGF (red) reaches 100% frequency in either the SR or SRTA cell population, then clonal expansion by a factor of *C* of that population occurs. **C**) PDGF overexpression leads to a potential transition to symmetric self-renewing cell division in TA cells (pink arrow). The rate of acquisition of self-renewal of a PDGF-overexpressing TA cell which has divided *k* times is given by *γ_k_* = max(*γ* −*kγ_step_*, 0), where *γ* is the rate of the most undifferentiated TA cell and *γ_step_* the factor by which this rate decreases per TA cell division. The first TA cell to become self-renewing exits the TA population and founds a new compartment of *N* self-renewing TA cells (gray oval). Every TA cell thereafter joins the new population, and, of the total cells in the compartment, *N* cells survive each time step. During each time step thereafter, a symmetric division occurs in this compartment wherein the offspring of one cell replaces another randomly chosen cell.

We classified genetic alterations into two distinct classes according to their mutational effects. Mutations in *INK4A/ARF*, *TP53*, and *NF1* were considered to result in increased proliferation. Since bi-allelic loss of *INK4A/ARF* and dominant negative *TP53* mutations lead to increased proliferative capabilities of cells [29], [30], the relative fitness (i.e. growth rate) of *INK4A/ARF^−/−^* and *TP53^+/−^* SR cells as compared to wild type cells are given by *R_ARF_*>1 and *R_TP53_*>1. Thus, in the SR cell niche, these mutant cells have a probability proportional to their relative fitness to be selected for cell division during each time step. Additionally, *INK4A/ARF^−/−^* and *TP53^+/−^* TA cells undergo *β_ARF_* and *β_TP53_* further cell divisions beyond the normal *z* divisions, respectively, before terminal differentiation (Fig. 2*A*). Since *INK4A/ARF* is a potent activator of TP53, the effects of alterations in these genes are not independent; we considered *R_ARF_* = *R_TP53_* and *β_ARF_* = *β_TP53_*, and also assumed that the fitness and additional divisions of cells carrying alterations in both genes is equal to those of cells carrying alterations in either gene alone [31]. Loss of *NF1* similarly leads to increased proliferative activity of cells if it arises on the background of either *TP53* or *INK4A/ARF* loss [32]; hence *INK4A/ARF^−/−^ NF1^−/−^* cells were considered to have relative fitness *R_ARF _*× *R_NF1,mut_* and divide a total of *z *+ *β_ARF _*+ *β_NF1_* times. In contrast, cells mutated in *NF1* without any other mutation have a fitness detriment, *R_NF1,wt_*≈0.05, and cannot undergo additional cell divisions beyond the normal *z* divisions [33].

In contrast, PDGF overexpression primarily causes expansion of SR cells as well as potential recruitment of other cells, and decreases the turnover time of cells [17], [34]. Hence we modeled the effect of PDGF overexpression in an SR cell cluster to be instantaneous expansion of the SR population and increase in the number of cell divisions per time step, both by a factor of *C* (Fig. 2*B*). Furthermore, PDGF-overexpressing non-self-renewing cells may acquire the ability to self-renew [16]. The rate of acquisition of self-renewal of TA cells that overexpress PDGF is given by *γ* – j*γ_step_, where *γ* represents the base rate of acquisition of self-renewal, *j* is the number of differentiating cell divisions the TA cell has undergone, and *γ_step_* is the level of decrease in self-renewal acquisition with differentiation. We chose to describe this effect through two parameters to encompass a decreasing probability of regaining self-renewal, resulting from either an increase in differentiation preventing self-renewal, or a loss of influence of microenvironmental factors as the TA cells leave the SVZ. The first TA cell to become self-renewing exits the TA population and founds a new niche of *CN* self-renewing TA (SRTA) cells (Fig. 2*C*). We referred to the event in which a PDGF-overexpressing TA cells acquires self-renewing properties and leaves the TA population as a ‘gamma event’. The initial gamma event leads to a new clone of *CN* SRTA cells, and every gamma event thereafter causes a TA cell to leave the TA population and enter the SRTA pool. These SRTA cells are self-renewing; however, we did not require that they recapitulate the full SR cell phenotype. The SRTA population then behaves in the same manner as the SR cell population, except that no asymmetric divisions can occur in this simplest version of the model; see Supporting Information S1 for a discussion of alternative assumptions and their effects.

We also considered two further non-mutational effects on the system. SR cells may occasionally produce two TA daughter cells [35], [36], [37], [38], a process we described by introducing a parameter λ into the system. With probability λ, an SR cell division produces one TA daughter cell and one SR daughter cell (asymmetric division), while with probability 1−λ, the SR cell division produces two TA daughter cells and no SR daughter cells. In order to maintain homeostasis, an additional symmetric SR cell division is then induced to replace the lost cell. Additionally, we considered the possibility of random cell death, where all cells have a probability *d* of undergoing apoptosis per time step. In the SR cell compartment, these deaths are compensated for by an additional cell division; in the TA cell population, another cell of the same maturity is chosen to divide again to replace the lost cell. The two replacement daughter cells are of the same maturity as the dead and divided cells.

We performed exact computer simulations of this stochastic process and also derived analytical approximations of the probabilities of cancer initiation from the different cell types (see Supporting Information S1 and Figs. S1 and S2). These approximations are useful for deriving predictions for parameter regimes for which the direct simulations would be computationally too expensive (Figs. S3).

To accurately determine the probability that a specific cell type serves as the cell of origin for PDGF- and NF1-driven gliomas, estimates of the system parameters are essential. We referred to investigations into the properties of glial cell lineages to obtain the most accurate estimates, but tested a range of values consistent with the literature to ensure the stability and robustness of our results; for those parameters for which an estimate had not been determined experimentally, we tested a large range of potential parameter values (see Supporting Information S1).

Let us now discuss the parameters in detail. The SVZ contains distinct cell clusters of 1–10 SR cells, each surrounded by ependymal cells [27]. We averaged over the range and estimated that the number of SR cells per niche is about *N*≈5. Due to lack of information about the rates of symmetric division of adult SR cells, we considered the rate of symmetric cell division of mature SR cells to be similar to division rates of radial glial cells. In order to maintain their self-renewing nature, the apical-basal polarity of these cells must be sustained [39]. Since the apical plasma membrane of SR cells consists of only 1–2% of the total plasma membrane, it was proposed [40] and later shown [39] that division of the apical plasma membrane governs symmetric versus asymmetric cell divisions. Since 40% of SR cells in the SVZ are dividing vertically and the remainder are dividing horizontally or obliquely [41], and roughly 50% of vertically dividing SR cells and none of the horizontally or obliquely dividing cells split the apical plasma membrane [39], the rate of symmetric SR cell division is *α*≈0.2. We also considered the possibility of a symmetric differentiation event in our model; however, the rates of such events are not known. We tested the predictions of our model for the frequencies of such events of 0, ½, and 1 and found that these changes had no effects on the relative contributions of different cell types to cancer initiation. *INK4A/ARF*, *TP53*, and *NF1* all have anti-proliferative effects [29], [30], [32] and therefore, inactivating mutations in these genes are expected to lead to increased growth as compared to wild type cells. Since both *INK4A/ARF* and *TP53* are in the same pathway [31], we considered the relative fitness of *INK4A/ARF* ^−/−^ and *TP53^+/−^* cells to be the same, *R_ARF_* = *R_TP53_*≈1.1; this choice was made since these cells show proliferation, but do not lead to a significant disruption of brain morphology [29]. However, the relative fitness values of *TP53* and *INK4A/ARF* need not be equal, and we tested for cases in which these choices vary. These values were tested over a range to establish the robustness of the predictions. Loss of *NF1*, in contrast, leads to a decreased fitness when inactivated without any cooperating genetic alterations, but to increased fitness in conjunction with *INK4A/ARF* or *TP53* loss [33]. This effect was addressed in the model by considering two fitness values – one denoting the fitness of *NF1^−/−^* cells, *R_NF1,wt_*≈0.05, and one denoting the fitness of *NF1^−/−^ TP53^+/−^* or *NF1^−/−^INK4A/ARF^−/−^* cells, *R_NF1,mut_*≈1.1. Fitness and proliferation effects of *INK4A/ARF* loss or *TP53* mutation in an *NF1* ^−/−^ cell are independent and so have a combined effect in double mutants; however, *INK4A/ARF* loss in combination with *TP53* mutation leads to no additional effects, as the same pathway is targeted by both alterations [31].

The number of symmetric divisions that glial TA cells can undergo before terminally differentiating is unknown. Therefore, we used data obtained for a different lineage of glial cells: O-2A progenitor cells. These cells were analyzed for the existence of a critical number of mitotic divisions before differentiating into non-dividing oligodendrocytes; using *in vitro* time lapse data coupled with a mathematical model, these cells were shown to require no more than two divisions before obtaining competence for differentiation [42]. After competence was achieved, we used an average of one additional division instead of using the branching process in Boucher et al. [42] to save on computation time. Thus we set *z*≈3, but also tested *z* = 1 and larger values. Additional proliferation of TA cells carrying *INK4A/ARF*, *TP53*, and *NF1* alterations was modeled as a fixed number of additional cell divisions *β_ARF_* = *β_TP53_* and *β_NF1_*, respectively, and initially, we set all three equal to 1. We considered only one additional division since there is no significant disruption of brain morphology in mice carrying those alterations, and so little gain in cell counts is expected [29]. *In vitro*, addition of PDGF to the medium results in immortalization and de-differentiation of oligodendrocyte progenitor cells and astrocytes [16]; similar effects were observed in cells with *INK4A/ARF* loss [43]. However, *in vivo* this effect has only been demonstrated in PDGF-overexpressing cells [19]. Thus, the potential for acquisition of self-renewing properties is most effectively conferred by overexpression of PDGF; we further assumed that the probability of a gamma event due to *INK4A/ARF* loss is small enough to be neglected. Since there are no estimates available for the rate at which gamma events occur, we tested a range of values. Similarly, as there may be margins of error on any estimate, we tested a wide range of potential values of all parameters around the estimates obtained from the literature and investigated the robustness of our predictions.

In response to PDGF overexpression, a 2-4-fold increase in the size of the SVZ is observed [44]; therefore, the expansion factor due to PDGF overexpression in the model is set to *C*≈3. The rate of cell division also increases about three-fold when PDGF is overexpressed [34], leading to an increase in the number of divisions per time step by a factor of *C* in our model. Somatic mutation rates in glial cell lineages have not been measured; instead, we used data from lymphoid cell assays as we expected that the mutation rate in the brain is similar. The spontaneous mutation rate per cell division per allele is approximately 0.5–3.5×10^−7^ [45], [46]. Thus, the mutation rate per allele per cell division of all genes was considered to be about *μ_PDGF_* = *μ_ARF_* = *μ_TP53_* = *μ_NF1_* = 1.0×10^−7^.

**Figure 3:**
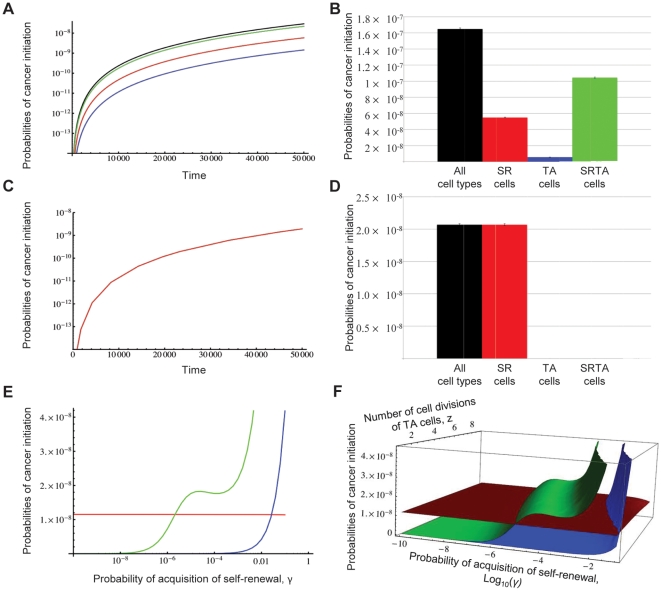
The most likely cell of origin of gliomas. **A**) Time course of the probability of PDGF-driven cancer initiation from SR cells (red), TA cells (blue), SRTA cells (green), and the total probability (black) for parameter values most accurately describing the human system. Parameter values are *α* = 0.2, *β* = 1, *C* = 3, *γ* = 0.005, *d* = 0.005 *γ_step_* = 0.0005, *μ_PDGF_* = 10^−7^, *μ_ARF_* = 10^−7^, *N* = 5, *R_ARF_* = 1.1, and *z* = 3. **B**) The most likely path to PDGF-driven cancer initiation for a comprehensive investigation of the parameter space. For each parameter value in the system, we define an interval spanning the likely values of the parameter (Table 1), and randomly choose combinations from the intervals. For the parameters *d*, *γ*, *μ_PDGF_*, and *μ_ARF_*, values are chosen from a log-uniform distribution while for all other parameters, values are chosen from a uniform distribution. This choice is repeated 1,000,000 times. The mean probabilities of cancer initiation from SR (red), TA (blue) and SRTA cells (green) are shown, along with the total probability (black) and the standard error. The probability of cancer initiation from SRTA cells is the dominant evolutionary trajectory towards brain cancer. **C**) Time course of the probability of NF1-driven cancer initiation from SR cells (red), TA cells (blue), and SRTA cells (green) for parameter values most accurately describing the human system, assuming that there is no gamma effect associated with NF1 loss. Parameter values are *α* = 0.2, *β_NF1_* = 1, *β_TP53_* = 1, *C* = 3, *d* = 0.005, *μ_NF1_* = 10^−7^, *μ_TP53_* = 10^−7^, *N* = 5, *R_TP53_* = 1.1, *R_NF1,wt_* = 0.2, *R_NF1,mut_* = 1.1, and *z* = 3. **D**) The most likely path to NF1-driven cancer initiation for a comprehensive investigation of the parameter space, assuming that there is no gamma effect associated with NF1 loss. For each parameter value in the system, we define an interval spanning the likely values of the parameter (Table 1), and randomly choose combinations from the intervals. For the parameters *d*, *γ*, *μ_NF1_*, and *μ_TP53_*, values are chosen from a log-uniform distribution while for all other parameters, values are chosen from a uniform distribution. This choice is repeated 1,000,000 times. The mean probabilities of cancer initiation from SR (red), TA (blue) and SRTA cells (green) are shown, along with the total probability (black) and the standard error. **E**) The probability of cancer initiation from SR cells (red), TA cells (blue), and SRTA cells (green) with different values of the rate at which TA cells acquire self-renewal, *γ*. **F**) The probability of cancer initiation from SR cells (red), TA cells (blue), and SRTA cells (green) with different values of the parameters *γ* and the number of cell divisions of TA cells, *z*.

When comparing the relative importance of the three populations (SR, TA, and SRTA cells) for cancer initiation, we found that the probability of cancer initiation from SRTA cells dominates the dynamics in the PDGF-induced case for almost all parameter choices (Figs. 3 and S3), while in the NF1-driven case, only SR cells can lead to cancer initiation (Fig. S4) if there is no appreciable gamma effect associated with either NF1 or TP53 loss. No such effect has been demonstrated for either of these alterations. However, loss of *NF1* was shown to transiently, but not permanently, promote self-renewal [14]. This effect may contribute to the recent finding that in a mouse model, NF1/TP53 deleted gliomas appeared to arise from non-stem cells [13]. In the context of our mathematical model, the differences between the respective animal studies [12], [13] can be accounted for by the differential ability of NF1 and/or TP53 to induce a (short-lived) gamma effect in cells such that under certain circumstances, a TA cell may be able to serve as the cell of origin for this subtype of glioma.

**Table 1:** Range of parameter values for the human brain.

PDGF-driven gliomas												
	*α*	*β_ARF_*	*C*	*D*	*γ_0_*	*γ_step_*	*μ_ARF_*	*μ_PDGF_*	*N*	*R_ARF_*	*Z*	*t*
Lower Bound	0.1	0	1	10^−4^	10^−5^	*γ_0_*/10	10^−8^	10^−8^	2	1.0	2	1,000
Upper Bound	0.3	3	5	10^−1^	10^−2^	*γ_0_*/5	10^−6^	10^−6^	15	1.3	5	50,000

NF1-driven gliomas												
	*α*	*β_NF1_*	*β_TP53_*	*D*	*μ_NF1_*	*μ_TP53_*	*N*	*R_NF1,wt_*	*R_NF1,mut_*	*R_TP53_*	*Z*	*t*
Lower Bound	0.1	0	0	10^−4^	10^−8^	10^−8^	2	0.01	1.0	1.0	2	1,000
Upper Bound	0.3	3	3	10^−1^	10^−6^	10^−6^	5	0.5	1.3	1.3	5	50,000

The table shows the ranges of parameter values used to calculate the most likely path to cancer initiation (see Figs. 3*B* and 3*D*) based on estimates from the literature (see main text for details and references). Note that in this case, we assume that there is no gamma effect associated with NF1 loss. See the main text for discussion of alternative assumptions.

For the mathematical model of PDGF-driven gliomas, we found that using those parameter values that most accurately describe the human brain results in the probability of cancer initiation from SRTA cells dominating the dynamics for all times (Fig. 3*A*). However, since many investigations were performed in the developing brain of rodents, there may be significant variation in parameter values when considering the human brain due to both interspecies differences and variability between developing and adult brain tissues. Thus, we performed a systematic analysis of the parameter space. We defined an interval around the estimates obtained from the literature that spans the likely values of each parameter (Table 1) and randomly chose sets of parameters from those intervals and examined the probability of cancer initiation from the various cell types. The mean probability for each cell type in PDGF-driven glioma along with the standard error is shown in Fig. 3*B*. Again, the probability of cancer initiation from SRTA cells is dominant in this analysis, followed by the probability of cancer initiation from SR cells. We repeated these tests for the NF1-driven case and again found that only SR cells can initiate tumorigenesis in this scenario (Fig. 3*C* and 3*D*) if no gamma effect is included for this case. As gamma increases from zero to small values, non-self-renewing cells can similarly serve as the cell of origin of this tumor subtype. Finally, we perturbed each parameter individually to study the robustness of the system to parameter variations, and found that only when the probability of de-differentiation and immortalization remains below 2 to 5×10^−5^, an SR cell is the most likely cell of origin (Fig. 3*E* and S3).

Of all the parameters analyzed in these mathematical modeling experiments, the rate at which TA cells acquire the potential to undergo symmetric self-renewing divisions, *γ*, is the most important determinant of the cell of origin (Fig. 3*E* and S3). Let us discuss the effects of this parameter with the example of PDGF-driven gliomas. If *γ* is very small (i.e., for our parameter regime, less than five in 10^5^ PDGF-overexpressing TA cells acquire self-renewing propensities per TA cell division), then a SR cell is the most likely cell of origin. However, if *γ* is of the order of 10^−4^ or larger, then a SRTA cell is the most likely cell of origin. We then explored the effect of the number of cell divisions TA cells can undergo, *z*, as well as *γ* on the probabilities of cancer initiation (Fig. 3*F*). Similarly, a threshold exists for values of *z* and *γ* that separates the dominant cell types for cancer initiation. When *z* increases, smaller values of *γ* are sufficient to shift the dominant cell type from SR to SRTA cells. In sum, unless *γ* is very small, an SRTA cell is the most likely cell of origin of PDGF-driven gliomas.

We next investigated the effects of therapeutic interventions on the probabilities of cancer initiation. We tested eight possible effects of treatments of PDGF-driven gliomas: (i) therapy that lowers gamma, thereby slowing the establishment and turnover of SRTA cells, (ii) treatments that decrease the expansion and recruitment of cells due to PDGF overexpression, (iii) treatments that selectively increase the death rate of mutant SR cells, (iv) mutant TA cells, (v) mutant SRTA cells, and (vi–viii) all combinations of increases of death rates of the three cell types. All other parameters are either infeasible to alter by therapy (such as mutation rates), would have little to no effect (such as relative fitness), or would severely disrupt the normal functioning of the brain – such as increasing the number of cells per niche or changing the relative rates of symmetric to asymmetric cell divisions. Treatments that lower the chance of self-renewal properties would be equivalent to lowering the parameter *γ* in the model, which would decrease the probability of cancer initiation in SRTA and TA cells (*X_R_* and *X_T_*), but not change the risk of cancer initiation from SR cells (Fig. S2 *E*, *F*, and *G*). A decrease in the expansion of PDGF-overexpressing cells is equivalent to lowering the value of *C* (Fig. S2 *C*). We found that a lower gamma, a lower *C*, and higher death rate of mutant SR cells lead to noticeable decreases in the probabilities of cancer initiation, while higher death rates of mutant TA cells have a negligible effect. Interestingly, higher death rates of mutant SRTA cells actually cause a significant increase in the risk of cancer initiation (Fig. S5). This increase is due to the SRTA population being formed of cells that are already mutated. Thus, any treatment that kills many, but not all, of the cells in this niche would cause increased turnover of cells and overall a faster accumulation of mutations. This effect becomes especially apparent with the administration of a treatment that increases death in SR and SRTA cells. A low death rate leads to a higher risk of cancer, since few mutations are lost by chance. As the death rate increases, there is a decrease in the probability of cancer initiation as more mutations are lost from the population. Once the death rate becomes too high, if a single mutant TA cell successfully becomes self-renewing and forms its own mutant SRTA cell niche, the increased turnover in this compartment allows more mutations to arise quickly and thereby enhances the probability of cancer initiation.

By contrast, there are relatively few effects that therapy can have on a glioma for which gamma is near zero. Since in that case, only SR cells can serve as the cell of origin, only those parameters that affect the SR cells cause a difference in the rate of cancer initiation. These parameters are *α*, *β_NF1_*, *β_TP53_*, *d*, *N*, *R_NF1,wt_*, and *R_NF1,mut_*. As with PDGF-driven gliomas, most of those parameters either have little to no effect or would significantly disrupt the brain structure if altered. The only parameter with an effect is the death rate, *d*. Contrary to the PDGF case, in a scenario with gamma near zero, increasing the death rate would increase the probability of cancer initiation (Fig. S2 *D*). This effect arises due to our modeling assumption that *TP53* decreases or prevents accidental death from occurring, thereby allowing further accumulation of mutations in cells wild type with respect to *TP53* while preventing loss of *TP53* mutated cells through accidental death.

We then turned to mouse modeling to validate the predictions of our mathematical framework that can be feasibly tested through biological experimentation. Note, however, that the mathematical model predicts the dynamics of tumor initiation rather than diagnosis and hence our predictions cannot directly be compared to cancer incidence data.

### Targeting PDGF to GFAP-expressing cells in different locations results in glioma formation

The experimental evidence regarding the cell of origin of NF1-driven gliomas remains controversial. In one series of experiments, genetic and stereotactic methods indicated that gliomas formed by combined loss of *NF1* and *TP53* and/or *PTEN* were restricted to neural stem/progenitor cells and were not initiated from cell types in the cortex or cerebellum [12], while in another experimental system, similar NF1/TP53 deleted gliomas appeared to arise from non-stem cells [13]. By contrast, there exists less experimental evidence for the PDGF-driven case. Recent evidence suggests that PDGF- and EGFR-induced gliomas may represent a distinct biology and cell of origin from other glioma subtypes [7], [8], [9], [11]. Furthermore, our mathematical model predicts that non-self-renewing cell types, specifically transit-amplifying cells, are very likely to initiate cancer in PDGF-induced gliomas. Therefore, we compared the extreme cases of PDGF-induced gliomagenesis from NSCs (as defined by GFAP positivity in the SVZ) versus reactive astrocytes (GFAP expression in the cortex, induced in response to injury by delivery of RCAS-PDGF) in order to determine if, when engineered into the mouse brain, it is possible to initiate cancer from a non-self renewing cell. We used the RCAS/tv-a system to target PDGF expression to GFAP-expressing cells of the SVZ, cortex and cerebellum. We injected *Gtv-a/ink4a/arf^−/−^* transgenic mice (where the *tv-a* receptor is expressed on GFAP-positive cells) with RCAS-PDGF using the stereotactic coordinates to target the SVZ (as a positive control for GFAP-expressing CNS stem cells), cortex and cerebellum. While these experiments did not directly compare transit amplifiers and neural stem/progenitor cells, they can confirm the ability of non-self renewing cells to initiate PDGF-induced gliomas, unlike the EGFR-induced case.

**Figure 4:**
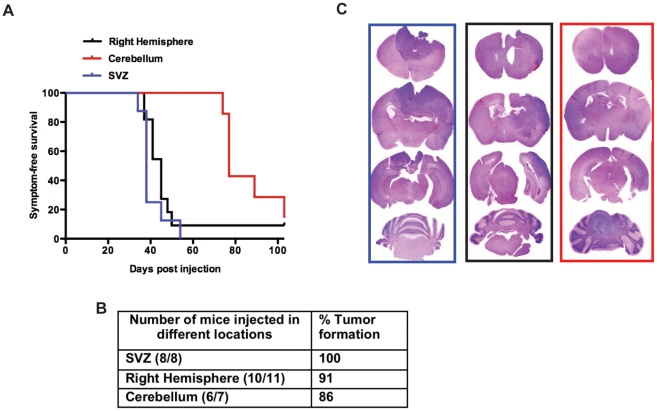
Overexpressing PDGFB in different locations of adult *Gtv-a*/*Ink4a-Arf^−/−^* mice leads to glioma formation. **A**) Kaplan-Meier survival curve of mice injected with PDGFB in different locations of *Gtv-a ink4a/arf^−/−^* mice demonstrated a tumorigenic advantage of the SVZ and right hemispheres versus the cerebellum. **B**) Table showing the number of mice injected tumor incidence (%) and median survival. **C**) Represent whole mount H&Es for corresponding groups in Kaplan-Meier survival curve in the boxes corresponding to the color of each group. Statistical analysis was performed to compare all the groups to the cerebellar group. All the mice without obvious evidence of tumors were sacrificed at 103 days post-injection.

Figure 4*A* depicts the Kaplan-Meier survival curve of mice injected in these three different locations. We found that all mice injected into the SVZ developed tumors. We also found that injection of RCAS-PDGF into the cortex resulted in 91% incidence of gliomas located in the lateral hemisphere with latency indistinguishable from the mice injected into the SVZ. The mice injected in the cerebellum showed 86% incidence of glioma formation, but exhibited a longer latency (Fig. 4*B*).

**Figure 5:**
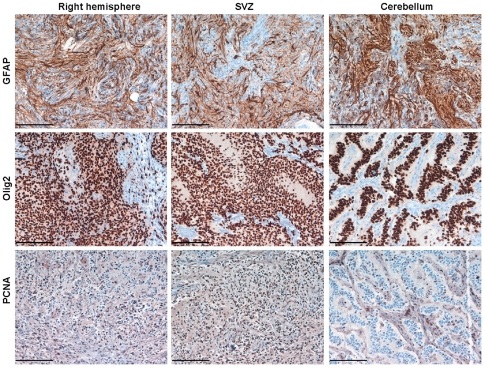
Histological characteristics of tumors generated in different locations of adult *Gtv-a ink4a/arf^−/−^* mice by overexpressing PDGFB. Representative images of immunostaining for GFAP, Olig2 shows that these tumors compose from regions more astrocytoma and others more oligodendroglioma histology. PCNA immunoreactivity shows similar level of positive cells in tumors generated by injecting PDGF in SVZ and right hemisphere and lower in cerebellum. Scale bars are 100 µm for all images.

Tumors initiated in all three locations demonstrated histological features of high-grade gliomas (Fig. 4*C*), including microvascular proliferation and in some cases extensive areas of pseudopalisading necrosis (Fig. 4*C*). All tumors generated were composed of regions with more oligo- and mixed astrocytoma histologies, with areas that were highly positive for GFAP and others positive for Olig2 (Fig. 5). PCNA staining of these tumors showed similar proliferation rates and was observed for tumors that arise from the SVZ and right hemisphere, and was lower for tumors arising in the cerebellum (Fig. 5).

In sum, the mouse modeling experiments demonstrated a nearly 100% incidence of PDGF-induced gliomas arising from GFAP-expressing cells in all three locations in the brain, with an equal latency between SVZ and cortex and a longer latency in the cerebellum. These results come in direct contrast to one of the previously published NF1 results [12], and verify that non-self renewing cells can potentially be the cell of origin of gliomas when a mutation conferring self-renewal, such as PDGF overexpression, occurs. However, this is the extent in which mouse modeling can determine the cell of origin. Mouse modeling can demonstrate which cell types are capable of serving as the cell of origin for tumors, but the results depend on the oncogene and experimental system used. Furthermore, mouse modeling can only address the propensity of a cell type to initiate gliomagenesis once a certain alteration or combination of alterations is engineered into the cell. It cannot address the relative likelihood of different cell types to initiate tumor development in the unperturbed system. As such, mouse modeling does not address issues of the evolutionary dynamics of mutations leading to tumorigenesis in humans. All of these caveats limit the conclusions that can be drawn from mouse modeling experiments regarding the cell of origin for tumors caused by spontaneous mutations.

## Discussion

In this paper, we have described a mathematical model of brain cancer development and determined the dominant evolutionary path towards cancer-initiating cells in two distinct subtypes of glioblastoma. Use of a mathematical model is necessary to determine the likelihood of a specific cell type to serve as the cell of origin of a tumor: while mouse modeling can show that certain mutations, introduced at a specific time and in a specific place, force a biological system to develop cancer, it cannot delineate how common such an emergence of a mutation may be. We designed our model to test how the consideration of a potential mechanism by which a transit-amplifying (TA) cell can become cancerous alters the likelihood of a neural stem cell (NSC) to initiate tumorigenesis. Using our model, we found that if one of the genetic alterations contributing to glioma formation allows a TA cell to undergo symmetric self-renewing divisions with a probability larger than a very low threshold, then the cell of origin is almost certainly a TA cell that has evolved this property. The risk of cancer initiation from a NSC, in comparison, is negligibly low in this situation. This scenario represents the PDGF-driven glioma subtype. In the case of gliomas induced by NF1 loss, the most likely cell of origin is determined by whether the initiating oncogenic events generate an appreciable gamma effect under the experimental conditions used. Thus, our mathematical model not only allows the prediction of the cell of origin for different glioma subtypes, but also helps to interpret recent conflicting findings about the origin of NF1-driven gliomas.

For our modeling purposes, we have defined the cell of origin as the cell that acquires the last mutation necessary since it is this cell that initiates cancer. Our mathematical modeling experiments also indicated that the cell that accumulates the first mutation towards glioma is almost always a NSC. This situation arises because a TA cell is unlikely to accumulate multiple mutations before terminally differentiating. In many cases, the cell harboring the first mutation will not accumulate the other alterations necessary for glioma formation; therefore, a consideration of the cell in which the last mutation arises is more meaningful. Alternatively to the terminology “cell of origin”, this cell may also be referred to as a cell permissive for tumorigenesis, while the non-tumorigenic forbearer may be called the cell in which the first oncogenic and ultimately initiating event arises. Both of these cells may be distinct from “tumor-propagating” cells, which maintain the tumor bulk [21]. The predictions of our model are robust with regard to changes in parameters (Fig. 3*B*) and with regard to variations in the structure of the model (see Supplementary Information S1 for more discussion). However, our model is restricted to consideration of glioblastoma subtypes in which the considered set of alterations arises: in a different subtype, other mechanisms may provide a greater impact on the dynamics of tumor initiation than those considered here. Nevertheless, should similar mechanisms be important for the development of other subtypes, our results would still hold. As more becomes known about the effects of genetic and epigenetic alterations driving tumorigenesis, our modeling approach will be useful to understand the dynamics of other systems.

When investigating the effects of therapeutic interventions on the probabilities of cancer initiation, we found that a lower rate at which self-renewal is acquired (gamma), a lower expansion factor due to PDGF-overexpression (*C*), and a higher death rate of mutated SR cells (*d*) decrease the risk of PDGF-driven glioma initiation, while in the case of NF1-driven gliomas, changes in SR cell characteristics (such as the fitness advantage conferred by loss of tumor suppressors) in addition to a potential gamma effect associated with NF1 loss can modify the chance of cancer initiation.

Due to the inability of mouse modeling to address the evolutionary dynamics of an unperturbed system, the predictions of our mathematical model concerning the relative likelihoods of each cell type to initiate tumorigenesis cannot be directly validated. However, the experimental system provides the possibility of partially validating the mathematical model by testing several conclusions of the mathematical framework: for example, we predicted that, if one of the genetic alterations driving tumorigenesis confers stem cell properties to cells, tumors do not necessarily have to emerge from stem cells but can arise from any cell type undergoing cell division. This finding is in accordance with our experimental evidence that PDGF-driven gliomas can originate in the SVZ, cortex and cerebellum if PDGF overexpression together with *INK4A/ARF* loss is engineered into the murine brain.

Assigning probabilities for specific cell types to serve as the cells of origin is more than an academic exercise. The cell of origin for the three major glioma subtypes may not be the same, and the different mutations required to transform these cells may ultimately lead to the differences in their biology. Multiple cell types have been shown to possess the potential to give rise to gliomas, but the properties of the genetic alterations that initiate the cancer phenotype greatly impact the probability of one cell type serving as cell of origin over the others. The property that matters the most is the rate of acquiring self-renewal, a parameter we term gamma. Gliomas driven by oncogenic mutations that have no appreciable gamma effect must arise from a NSC. Thus, other combinations of alterations that do not result in a gamma effect must also arise in a NSC. By contrast, because of the significant gamma effect associated with dysregulated PDGF signaling, gliomas induced by PDGF can originate in nearly any cell including NSC, TA, OPCs and reactive astrocytes. However, mathematical modeling determines that the most likely cell of origin of PDGF-driven gliomas due to sporadic mutation is a transit-amplifying cell. This interdisciplinary approach of mathematical and mouse modeling not only allows us to investigate the results of introducing mutations into different cell types *in vivo*, but also to determine the likelihood that individual cell types serve as the cells of origin of gliomas in an unperturbed system.

## Methods

### Mice

*Gtv-a ink4a/arf^−/−^* mice were used at an age ranging from 5 to 13 weeks for all experiments. Animal experiments were conducted using protocols approved by the Institutional Animal Care and Use Committees of Memorial Sloan-Kettering Cancer Center. The approved protocol number is 00-11–189 (MSKCC, last approved date is 3/15/2010).

### Cell culture and transfections

DF-1 cells were purchased from ATCC. Cells were grown at 39°C according to ATCC instructions. Transfections with RCAS-PDGFB-HA were performed using Fugene 6 transfection kit (Roche # 11814443001) according to manufacturer's instruction.

### Intracranial injections

Injections were performed using stereotactic fixation device (Stoelting, Wood Dale, IL). Mice used for these experiments were adult from 5 to 13 weeks old. Mice were anaesthetized with i.p. injections of ketamine (0.1 mg/g) and xylazine (0.02 mg/g). 1 µl of 4×10^4^ transfected DF-1 cell suspension was delivered using 30-gauge needle attached to Hamilton syringe. Locations were determined according to the instructions in atlas [47]. Coordinates for SVZ injections were: AP-0 mm from bregma, Lat-1,2 mm (right of midline), and a depth-1.5 mm from the dural surface. Right hemisphere: AP-0 mm from bregma, Lat-3.0 mm (right) depth-1.0 mm from dural surface. Cerebellum: AP -5.5 mm from bregma, Lat-1.5 mm (to the left), depth-1.0 mm from the dural surface. Mice were monitored carefully and sacrificed when they displayed symptoms of tumor development (lethargy, head tilt). Experiments were stopped at 103^rd^ day.

### Tissue processing

Animals used for histological analysis were sacrificed, and brains were removed and fixed in 10% neutral buffered formalin for 72 h. Fixed tissues were then embedded in paraffin. Formalin-fixed, paraffin-embedded specimens were serially sectioned and slide mounted. The sections were deparaffinized in histo-clear (Richard-Allan Scientific) and were passed through graded alcohols before staining with H&E reagent.

### Immunohistochemistry

An automated staining processing (Discovery-XT, Ventana Medical Systems, Inc.) was used for immunohistochemical detection. The protocols were established at the Molecular Cytology Core Facility and at Brain Tumor Center at MSKCC. Anti-PCNA antibodies were obtained from DAKO (# MO879), anti-GFAP from DAKO (Z0344) and anti-Olig2 from Millipore (AB9610), and used at 1/2000, 1/8000 and 1/250 dilutions in 2% BSA in PBS correspondingly.

## Supporting Information

Figure S1 **Fit of the analytical approximation to simulation results for PDGF-driven gliomas.** We investigate the fit of the analytical approximation derived for the PDGF-driven case with the output of exact stochastic computer simulations while varying each parameter and keeping the other values constant. Dots represent the results of the exact stochastic computer simulations and curves represent the results of the analytical approximation. The black curve shows the total probability of cancer initiation, the red curve the probability of cancer initiation from self-renewing (SR) cells, the blue curve the probability of cancer initiation from transit-amplifying (TA) cells, and the green curve the probability of cancer initiation from self-renewing transit-amplifying (SRTA) cells. The standard parameter values are *α* = 0.2 (probability of a symmetric SR cell division); *β_ARF_* = 1 (additional number of cell divisions of *INK4A/ARF^−/−^* TA cells); *C* = 3 (expansion factor due to PDGF overexpression); *d* = 0.005 (per cell per division accidental death rate); *γ* = 0.005 (rate of acquisition of self-renewal in the most undifferentiated PDGF-overexpressing TA cells); *γ_steo_* = 0.0005 (reduction factor of *γ* with each cell division); *μ_ARF_* = *μ_PDGF_* = 10^−4^ (mutation rate per allele); *N* = 5 (number of SR cells); *R_ARF_* = 1.1 (relative fitness value (i.e. growth rate) of *INK4A/ARF^−/−^* SR cells); *z* = 3 (number of TA cell divisions);, and *t* = 4000 (time).
(TIF)

Figure S2 **Fit of the analytical approximation to simulation results for NF1-driven gliomas.** We investigate the fit of the analytical approximation in the PDGF-driven case with the output of exact stochastic computer simulations while varying each parameter and keeping the other values constant. Dots represent the results of the exact stochastic computer simulations and curves represent the results of the analytical approximation. The red curve shows the probability of cancer initiation from self-renewing (SR) cells; all other cell types are zero and so are not displayed. Note that this latter effect arises since we assume that there is no appreciable gamma effect associated with NF1 loss; see the main text for discussion of alternative assumptions. The standard parameter values are *α* = 0.2 (probability of a symmetric SR cell division); *β_NF1_* = *β_TP53_* = 1 (additional number of cell divisions of *NF1^−/−^* and *TP53*-dominant negative TA cells); *d* = 0.005 (per cell per division accidental death rate); *μ_NF1_* = *μ_TP53_* = 10^−4^ (mutation rate per allele); *N* = 5 (number of SR cells); *R_NF1,wt_* = 0.2 (relative fitness value (i.e. growth rate) of *NF1^−/−^* mutant SR cells without *TP53* or *INK4A/ARF* mutations); *R_NF1,mut_* = 1.1 (relative fitness value (i.e. growth rate) of *NF1^−/−^* mutant SR cells with *TP53* or *INK4A/ARF* mutations); *R_TP53_* = 1.1 (relative fitness value (i.e. growth rate) of *TP53* dominant negative SR cells); *z* = 3 (number of TA cell divisions), and *t* = 4000 (time).
(TIF)

Figure S3 **Parameter dependence of the probabilities of cancer initiation for PDGF-driven gliomas using mutation rates of human cells.** We investigate the parameter dependence of the probabilities of cancer initiation using the differential equation systems in the PDGF-driven case by varying each parameter while keeping the other values constant. The red curve shows the probability of cancer initiation from self-renewing (SR) cells, the blue curve the probability of cancer initiation from transit-amplifying (TA) cells, and the green curve the probability of cancer initiation from self-renewing transit-amplifying (SRTA) cells. Parameters are kept the same as when fitting the approximation to simulation (Fig. S1), but the default mutation rate is decreased to *μ_ARF_* = *μ_PDGF_* = 10^−7^.
(TIF)

Figure S4 **Parameter dependence of the probabilities of cancer initiation for PDGF-driven gliomas when symmetric differentiation is introduced.** We investigate the parameter dependence of the probabilities of cancer initiation in the NF1-driven case by varying each parameter while keeping the other values constant. The red curve shows the probability of cancer initiation from self-renewing (SR) cells, the blue curve the probability of cancer initiation from transit-amplifying (TA) cells, and the green curve the probability of cancer initiation from self-renewing transit-amplifying (SRTA) cells. Parameters are kept the same as when fitting the approximation to simulation (Fig. S1), but only asymmetric differentiation (solid lines), half symmetric differentiation steps and half asymmetric differentiation steps (dashed lines), and all symmetric differentiation steps (dotted lines) are displayed. These results are derived from the exact stochastic computer simulations.
(TIF)

Figure S5 **Effect of therapeutic interventions that increase the death rates in SR, TA, and SRTA cells, and all combinations thereof on PDGF-driven gliomagenesis.** We investigate the effect of treatments that selectively increase cell death in **A**) SR cells, **B**) TA cells, **C**) SRTA cells, **D**) SR and TA cells, **E**) SR and SRTA cells, and **F**) TA and SRTA cells. The red curve shows the probability of cancer initiation from self-renewing (SR) cells, the blue curve the probability of cancer initiation from transit-amplifying (TA) cells, and the green curve the probability of cancer initiation from self-renewing transit-amplifying (SRTA) cells. All parameters are kept the same as in Figure S1 except for death rates.
(TIF)

Supporting Information S1 (PDF)
