# Supplementary figures and images for: The Probable Cell of Origin of NF1- and PDGF-Driven Glioblastomas
Source: PLoS One. 2011 Sep 9;6(9):e24454. doi: 10.1371/journal.pone.0024454 (PMC3170338; doi:10.1371/journal.pone.0024454)

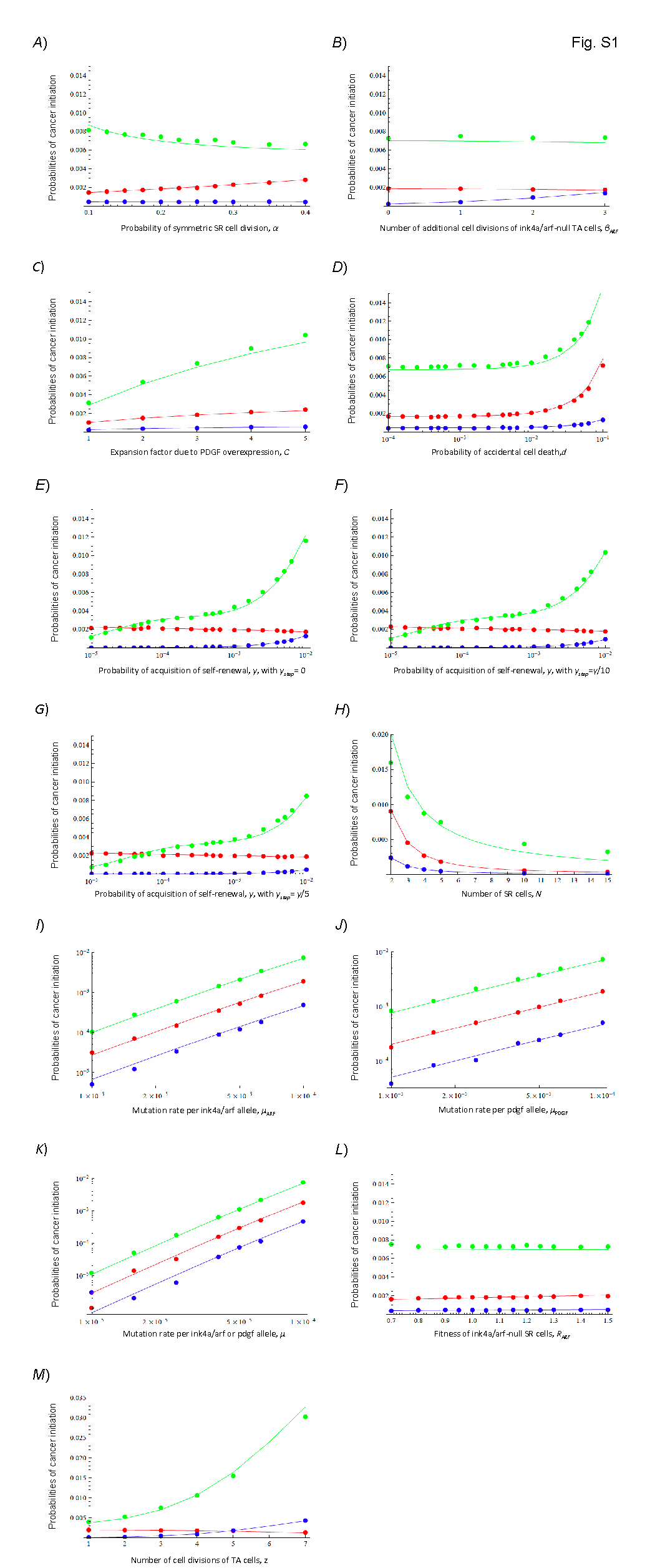

Supplement: Figure S1 — Fit of the analytical approximation to simulation results for PDGF-driven gliomas. We investigate the fit of the analytical approximation derived for the PDGF-driven case with the output of exact stochastic computer simulations while varying each parameter and keeping the other values constant. Dots represent the results of the exact stochastic computer simulations and curves represent the results of the analytical approximation. The black curve shows the total probability of cancer initiation, the red curve the probability of cancer initiation from self-renewing (SR) cells, the blue curve the probability of cancer initiation from transit-amplifying (TA) cells, and the green curve the probability of cancer initiation from self-renewing transit-amplifying (SRTA) cells. The standard parameter values are α = 0.2 (probability of a symmetric SR cell division); βARF = 1 (additional number of cell divisions of INK4A/ARF−/− TA cells); C = 3 (expansion factor due to PDGF overexpression); d = 0.005 (per cell per division accidental death rate); γ = 0.005 (rate of acquisition of self-renewal in the most undifferentiated PDGF-overexpressing TA cells); γsteo = 0.0005 (reduction factor of γ with each cell division); μARF = μPDGF = 10−4 (mutation rate per allele); N = 5 (number of SR cells); RARF = 1.1 (relative fitness value (i.e. growth rate) of INK4A/ARF−/− SR cells); z = 3 (number of TA cell divisions);, and t = 4000 (time). (TIF) [file pone.0024454.s001.tif]

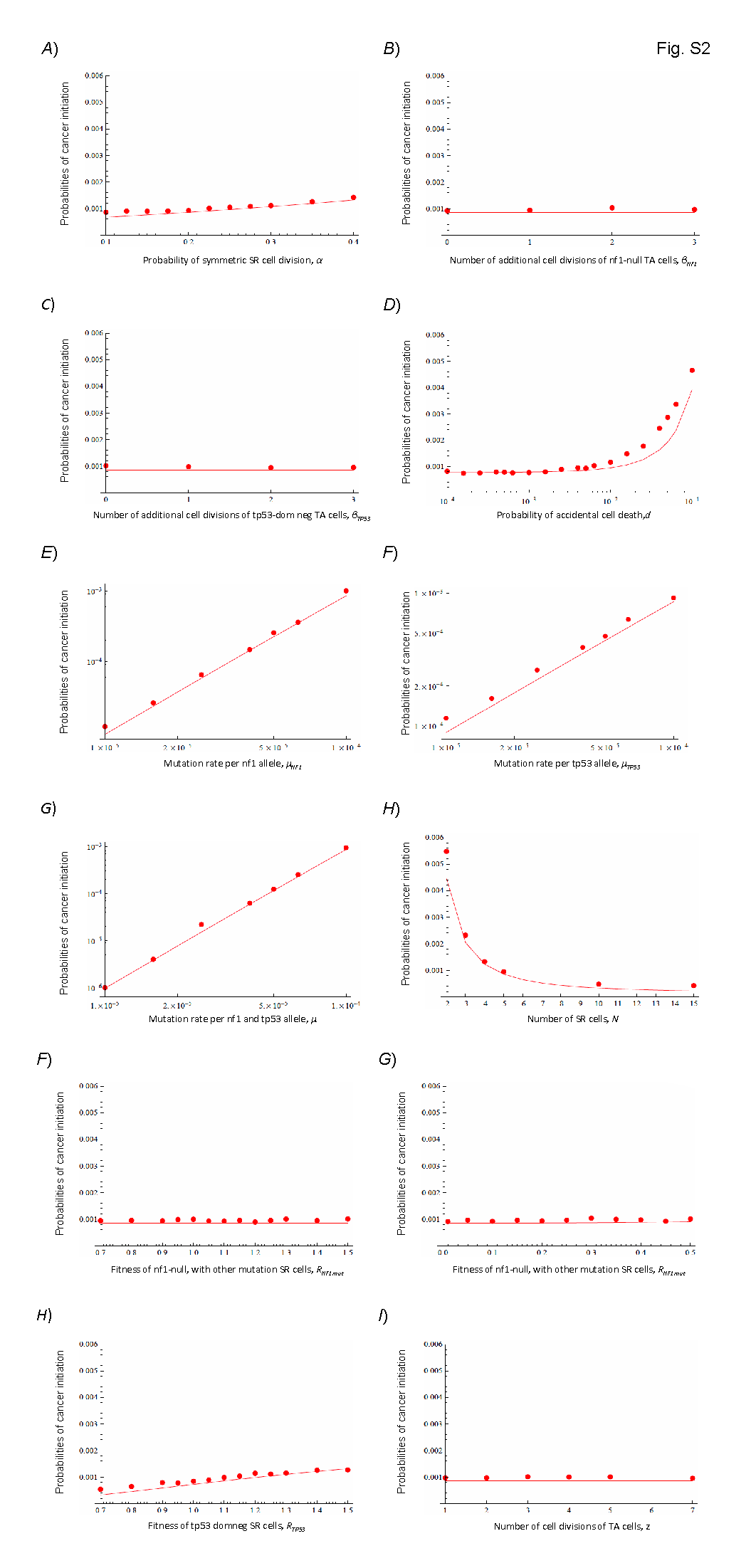

Supplement: Figure S2 — Fit of the analytical approximation to simulation results for NF1-driven gliomas. We investigate the fit of the analytical approximation in the PDGF-driven case with the output of exact stochastic computer simulations while varying each parameter and keeping the other values constant. Dots represent the results of the exact stochastic computer simulations and curves represent the results of the analytical approximation. The red curve shows the probability of cancer initiation from self-renewing (SR) cells; all other cell types are zero and so are not displayed. Note that this latter effect arises since we assume that there is no appreciable gamma effect associated with NF1 loss; see the main text for discussion of alternative assumptions. The standard parameter values are α = 0.2 (probability of a symmetric SR cell division); βNF1 = βTP53 = 1 (additional number of cell divisions of NF1−/− and TP53-dominant negative TA cells); d = 0.005 (per cell per division accidental death rate); μNF1 = μTP53 = 10−4 (mutation rate per allele); N = 5 (number of SR cells); RNF1,wt = 0.2 (relative fitness value (i.e. growth rate) of NF1−/− mutant SR cells without TP53 or INK4A/ARF mutations); RNF1,mut = 1.1 (relative fitness value (i.e. growth rate) of NF1−/− mutant SR cells with TP53 or INK4A/ARF mutations); RTP53 = 1.1 (relative fitness value (i.e. growth rate) of TP53 dominant negative SR cells); z = 3 (number of TA cell divisions), and t = 4000 (time). (TIF) [file pone.0024454.s002.tif]

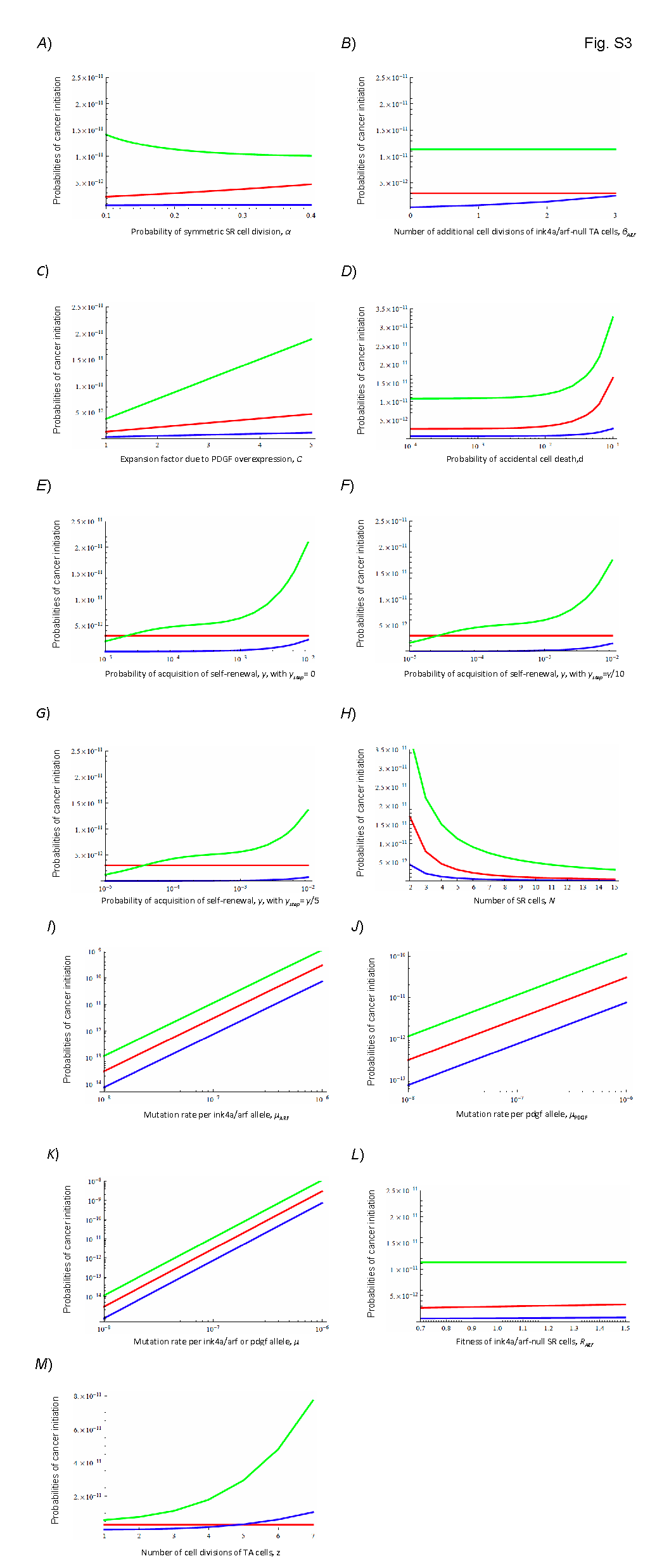

Supplement: Figure S3 — Parameter dependence of the probabilities of cancer initiation for PDGF-driven gliomas using mutation rates of human cells. We investigate the parameter dependence of the probabilities of cancer initiation using the differential equation systems in the PDGF-driven case by varying each parameter while keeping the other values constant. The red curve shows the probability of cancer initiation from self-renewing (SR) cells, the blue curve the probability of cancer initiation from transit-amplifying (TA) cells, and the green curve the probability of cancer initiation from self-renewing transit-amplifying (SRTA) cells. Parameters are kept the same as when fitting the approximation to simulation (Fig. S1), but the default mutation rate is decreased to μARF = μPDGF = 10−7. (TIF) [file pone.0024454.s003.tif]

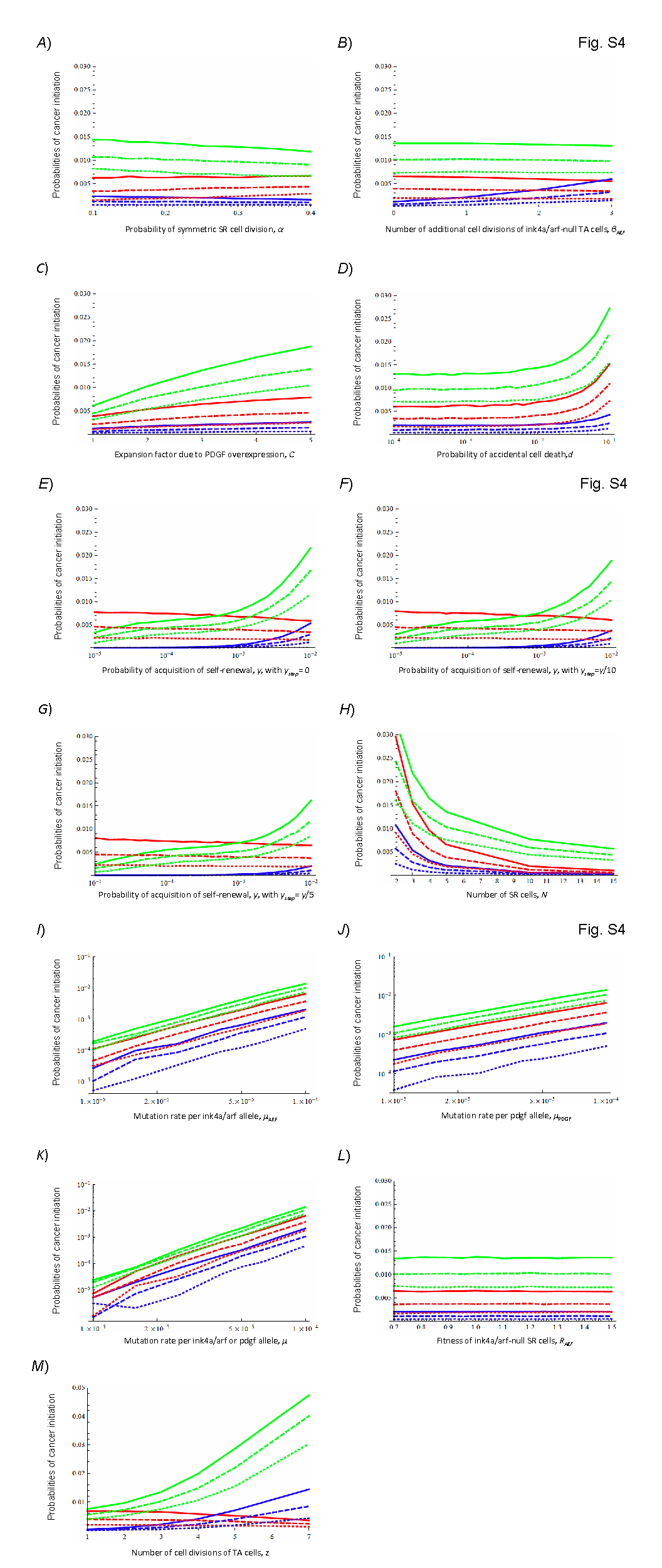

Supplement: Figure S4 — Parameter dependence of the probabilities of cancer initiation for PDGF-driven gliomas when symmetric differentiation is introduced. We investigate the parameter dependence of the probabilities of cancer initiation in the NF1-driven case by varying each parameter while keeping the other values constant. The red curve shows the probability of cancer initiation from self-renewing (SR) cells, the blue curve the probability of cancer initiation from transit-amplifying (TA) cells, and the green curve the probability of cancer initiation from self-renewing transit-amplifying (SRTA) cells. Parameters are kept the same as when fitting the approximation to simulation (Fig. S1), but only asymmetric differentiation (solid lines), half symmetric differentiation steps and half asymmetric differentiation steps (dashed lines), and all symmetric differentiation steps (dotted lines) are displayed. These results are derived from the exact stochastic computer simulations. (TIF) [file pone.0024454.s004.tif]

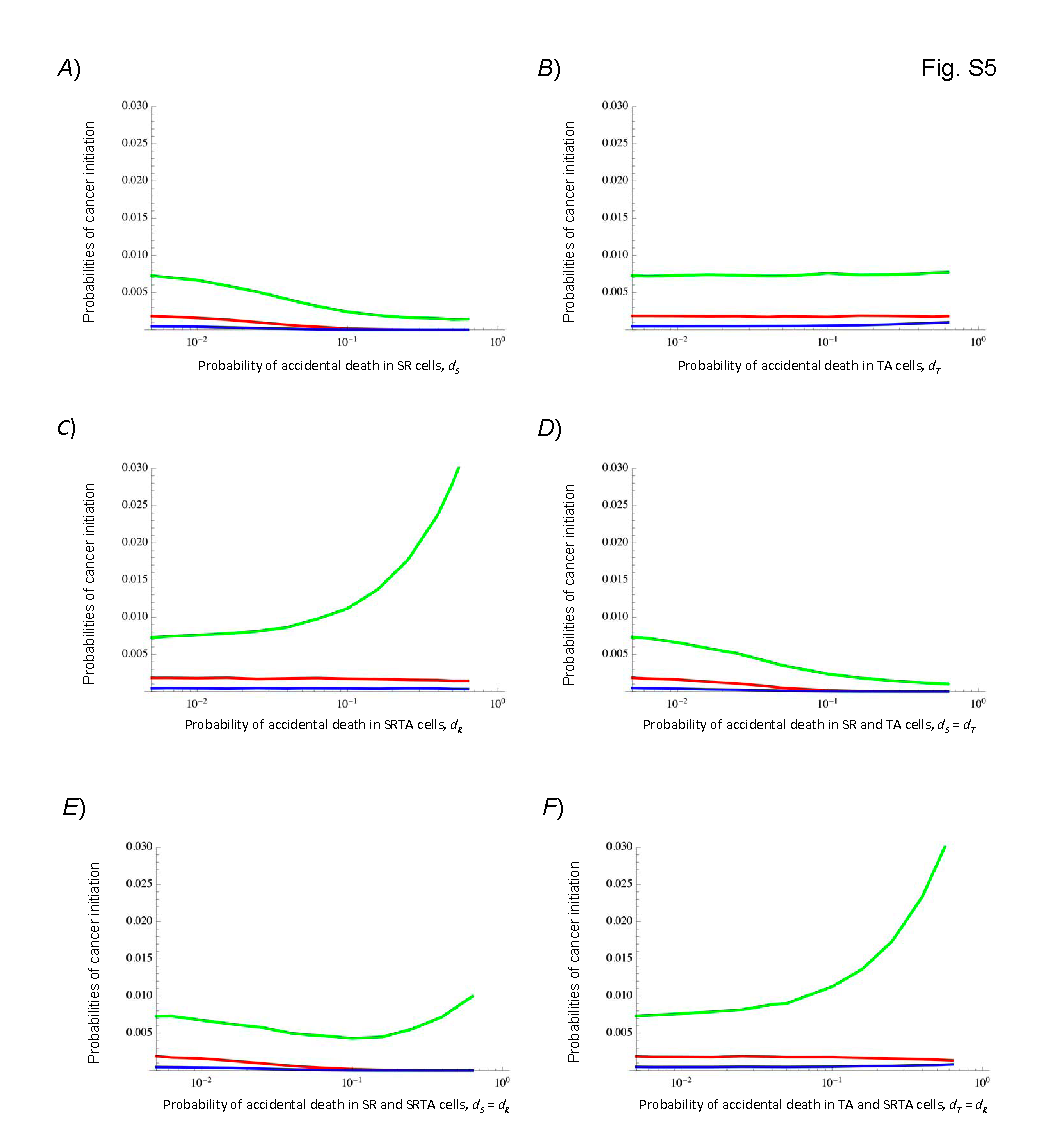

Supplement: Figure S5 — Effect of therapeutic interventions that increase the death rates in SR, TA, and SRTA cells, and all combinations thereof on PDGF-driven gliomagenesis. We investigate the effect of treatments that selectively increase cell death in A) SR cells, B) TA cells, C) SRTA cells, D) SR and TA cells, E) SR and SRTA cells, and F) TA and SRTA cells. The red curve shows the probability of cancer initiation from self-renewing (SR) cells, the blue curve the probability of cancer initiation from transit-amplifying (TA) cells, and the green curve the probability of cancer initiation from self-renewing transit-amplifying (SRTA) cells. All parameters are kept the same as in Figure S1 except for death rates. (TIF) [file pone.0024454.s005.tif]
