# Supplementary material for: The Probable Cell of Origin of NF1- and PDGF-Driven Glioblastomas
Source: PLoS One. 2011 Sep 9;6(9):e24454. doi: 10.1371/journal.pone.0024454 (PMC3170338; doi:10.1371/journal.pone.0024454)
Supplement: Supporting Information S1 — (PDF) [file pone.0024454.s006.pdf]

Supplemental Information

Inventory:

1. Supplemental Experimental Procedures
2. Supplemental References
3. Supplemental Tables
4. Supplemental Figures

Supplemental Experimental Procedures

We designed a stochastic mathematical model of gliomagenesis to identify the most likely cell of origin of PDGF- and NF1-induced gliomas. This model is part of a growing body of literature devoted to the mathematical modeling of glioma growth [1,2,3,4,5,6,7,8,9,10,11,12].

We performed exact stochastic computer simulations of the system (for a full description of the system, see the main text) and derived analytical approximations of the probabilities of cancer initiation from the different cell types. These analytical approximations are useful to analyze parameter regimes for which stochastic simulations would be computationally too expensive. We considered multiple independent cell clusters or niches of neural stem and progenitor cells within the brain [13]. We investigated the dynamics of one niche within the brain since the total probability of cancer initiation is given by the probability per niche times the number of niches; hence, a consideration of all niches does not alter the most likely cell of origin of brain cancer. We derived equations for the PDGF and NF1 cases individually. Let us first discuss the case of PDGF-driven gliomas; we will consider NF1-driven gliomas in a later section.

In the PDGF-driven case, we consider a population of N self-renewing (SR) cells, one of which divides each time step. This division can be self-renewing symmetric with probability α, in which case another SR cell is chosen to die to maintain homeostasis in the tissue, or differentiating with probability $1-\alpha$. With probability λ, the division resulting in differentiation is asymmetric, in which case one of the daughter cells remains a SR cell while the other becomes

a transit amplifying (TA) cell. The remaining fraction 1 – λ differentiate symmetrically, producing two TA cells and causing a second SR cell to divide symmetrically to maintain homeostasis. Since the inclusion of symmetric differentiation did not change the relative ordering of the probabilities of cancer initiation between cell types (Table S4), we set λ = 1 and neglected it in the differential equation system for mathematical simplicity.

Each TA cell divides symmetrically, producing more differentiated cells with each division, for a total of z times before terminal differentiation and loss from the system. Additional accidental cell deaths can also occur, in which case a cell of equal maturity will divide to replace it. Genetic alterations can arise in one of the daughter cells of any division. We denote the mutation rate per allele per division by μ_{ARF} and μ_{PDGF} for alterations leading to *INK4A/ARF* inactivation and those leading to PDGF overexpression, respectively. Bi-allelic inactivation of *INK4A/ARF* results in an increased growth rate (i.e. relative fitness) of $R_{ARF} > 1$ in SR cells and an additional β_{ARF} cell divisions in TA cells. PDGF overexpression in 100% of cells in the SR cell population results in expansion by a factor of C of that population and also C SR cell divisions per time step. In TA cells, PDGF overexpression confers a probability of regaining self-renewal capabilities γ_i, which is dependent on the differentiation level i of the cell which overexpresses PDGF. When the first PDGF-overexpressing TA cell becomes self-renewing, a new population of self-renewing transit amplifying (SRTA) cells of size CN is created. Cancer is initiated when a self-renewing cell with loss of both *INK4A/AR* alleles and overexpression of PDGF has emerged. This cell can arise from an SR or SRTA cell, or alternatively from a TA cell which has undergone a gamma event.

Let us first consider the dynamics of SR and SRTA cells. Thus, we use a state space consisting of all possible combinations of mutations in SR and SRTA cell populations. Since we consider the number of SR cells, N, in each niche to be small [13], the mean waiting time until the appearance of a mutated cell is much longer than the average time for fixation of its lineage. Hence we can describe the evolutionary dynamics as a simple Markovian jump between populations consisting only of a single type of SR cells; the dynamics of SRTA cells are treated similarly. There are five states of SR cells and three states of SRTA cells before the generation of the first cancer-initiating cell. Thus, we have fifteen states before the emergence of a cancer-

initiating clone. For each cell type, there also exists a state in which the cancer-initiating cell has already emerged. The states are enumerated as in Table S1.

Denote by X_{ij} the probability that the SR and SRTA cell populations are in states i and j, and by X_S, X_T, and X_R the probabilities that a cancer-initiating clone arises from an SR, TA, and SRTA cell, respectively. Let us first consider only the dynamics within the SR cell population, given by

$$\dot{X}_{0j} = -2\left(\alpha + \frac{1-\alpha}{2} + Nd\right)\left\{\mu_{PDGF} + \mu_{ARF}\right\}X_{0j},$$

$$\dot{X}_{1j} = 2\left(\alpha + \frac{1-\alpha}{2} + Nd\right)\left\{\mu_{PDGF}X_{0j} - \mu_{ARF}X_{1j}\right\},$$

$$\dot{X}_{2j} = \left(\alpha + \frac{1-\alpha}{2} + Nd\right)\left\{2\mu_{ARF}X_{0j} - \left(2\mu_{PDGF} + N\rho\left(R_{ARF}\right)\mu_{ARF}\right)X_{2j}\right\}, \qquad \text{for all } j \in (0,1,3)$$

$$\dot{X}_{3j} = \left(\alpha + \frac{1-\alpha}{2} + Nd\right)\left\{2\mu_{PDGF}X_{2j} + 2\mu_{ARF}X_{1j} - CN\mu_{ARF}X_{3j}\right\},$$

$$\dot{X}_{4j} = \left(\alpha + \frac{1-\alpha}{2} + Nd\right)N\left\{\rho\left(R_{ARF}\right)\mu_{\mathrm{ARF}}X_{2j} - 2\mu_{PDGF}X_{4j}\right\},$$

$$\dot{X}_{S} = \left(\alpha + \frac{1-\alpha}{2} + Nd\right)N\sum_j\left(C\mu_{ARF}X_{3j} + 2\mu_{PDGF}X_{4j}\right).$$

Here α represents the probability of a symmetric SR cell division. When an asymmetric SR cell division occurs and a mutation emerges, this mutation is retained in the SR cell population with probability ½. Additionally, each accidental death is compensated with a symmetric division to maintain homeostasis. This event can be treated as an additional Moran step, potentially allowing for a mutation to arise. Thus, the probability that SR cells accumulate a mutation per division is expressed as (α + (1 − α)/2 + Nd) or (α + (1 − α)/2 + CNd)*[mutation rate], which is dependent on the number of cells in the SR population. In these equations, we have

$$\rho\left(R_{ARF}\right) = \frac{1 - \frac{1}{R_{ARF}}}{1 - \frac{1}{R_{ARF}{}^{N}}},$$

where R_{ARF} is the relative growth rate of *INK4A/ARF*$^{-/-}$ SR cells. Time is measured in units of N SR cell divisions and initially, all SR cells are unmutated (X_{00} = 1, X_{ij} = 0 for all i and j). Note that state S is the only state in which mutated SR cells need not take over the population since we assume that the first SR cell with that genotype initiates clonal expansion with probability one.

Let us next consider the dynamics of TA cells. Since TA cells are generated by asymmetric SR cell divisions, the initial mutational state of the TA compartment depends on the state of the SR cell compartment. For example, if the most undifferentiated TA cell is unmutated, then its lineage needs to accumulate at least three alterations (an alteration leading to PDGF overexpression and mutations inactivating both alleles of *INK4A/ARF*) and a gamma event (i.e. the acquisition of self-renewal) to generate a cancer-initiating cell. However, if a TA already harbors a mutation leading to PDGF overexpression, then its lineage needs to accumulate only two further mutations and a gamma event. In the derivation of the analytical approximation, we ignore the cases in which TA cells need to accumulate more than one mutation before terminally differentiating since the probability of these cases occurring is very small. The cell of origin is then defined as the cell that accumulates the last mutation necessary for cancer initiation.

Let us consider the case in which a lineage of TA cells needs to accumulate only one mutation and a gamma event to produce a cancer-initiating cell. These lineages consist of the populations of TA cells arising from states 3 and 4 in the SR cell compartment (see Table S1). The probabilities that a cancer-initiating clone is produced from these lineages per time unit are respectively given by

$$F_{ARF} = \frac{1}{2}(1+2d)\mu_{ARF} \sum_{k=0}^{z} \sum_{l=k}^{z+\beta_{ARF}} 2^l (\gamma - l\gamma_{step})$$

for state 3, and

$$F_{PDGF} = \frac{1}{2}(1+2d)\mu_{PDGF} \sum_{k=0}^{z+\beta_{ARF}} \sum_{l=k}^{z+\beta_{ARF}} 2^l (\gamma - l\gamma_{step})$$

for state 4.

The chance that a TA clone accumulates two events before terminally differentiating was derived in Haeno et al. [14]. These rates of cancer-initiation from a TA compartment are used to derive the dynamics of TA cells,

$$\dot{X}_{3j} = -(1-\alpha)CNF_{ARF}X_{3j},$$

$$\dot{X}_{4j} = -(1-\alpha)NF_{PDGF}X_{4j}, \qquad \text{for all } j \in (0,1,3)$$

$$\dot{X}_{T} = (1-\alpha)N\sum_j \left(CF_{ARF}X_{3j} + F_{PDGF}X_{4j}\right).$$

These quantities contain a factor of N since time is measured in units of N SR cell divisions.

Let us now consider the probability of cancer initiation from TA cells that have acquired self-renewing propensities before accumulating the final mutation (SRTA cells). The difference between the dynamics of the SR and SRTA cell populations is that (i) the latter cells always undergo symmetric self-renewing cell divisions, (ii) at the beginning, there is no population of SRTA cells, and (iii) the SRTA population never contains unmutated cells. The latter situation arises since we assume that PDGF overexpression is required for cells to experience a gamma event.

The dynamics within the SRTA population ignoring gamma events is given by

$$\dot{X}_{i1} = -(1+Nd)2\mu_{ARF}X_{i1},$$

$$\dot{X}_{i3} = (1+Nd)\left\{2\mu_{ARF}X_{i1} - CN\mu_{ARF}X_{i3}\right\}, \qquad \text{for all } i \in (0,1,2,3,4)$$

$$\dot{X}_{R} = (1+Nd)CN\mu_{ARF}\sum_i X_{i3}.$$

Denote by G_p the rate at which TA cells overexpressing PDGF experience a gamma event; it is given by

$$G_p = \sum_{k=0}^{z} 2^k\left(\gamma - k\gamma_{step}\right).$$

A special scenario arises when a mutation leading to PDGF overexpression arises in the TA cell population, and such a cell acquires self-renewal before the clone terminally differentiates. The rates at which such a gamma event occurs is given by

$$G_{PDGF} = \frac{1}{2}(1+2d)\mu_{PDGF} \sum_{k=0}^{z} \sum_{l=k}^{z} 2^l \left(\gamma - l\gamma_{step}\right).$$

Similarly, a PDGF-overexpressing TA cell may evolve a mutation in *INK4A/ARF*, after which one of its offspring experiences a gamma event before the clone terminally differentiates. The rate at which such a gamma event occurs is given by

$$G_{ARF} = \frac{1}{2}(1+2d)\mu_{ARF} \sum_{k=0}^{z} \sum_{l=k}^{z} 2^l \left(\gamma - l\gamma_{step}\right).$$

Then the dynamics of SR and SRTA cells due to gamma events is given by

$$\dot{X}_{00} = -(1-\alpha)NG_{PDGF}X_{00},$$

$$\dot{X}_{01} = (1-\alpha)NG_{PDGF}\left\{X_{00} + X_{03}C^{-1}N^{-1}\right\},$$

$$\dot{X}_{03} = -(1-\alpha)G_{PDGF}X_{03}C^{-1},$$

$$\dot{X}_{10} = -(1-\alpha)\left\{1 - \exp[-CNG_p] + CNG_{ARF}\right\}X_{10},$$

$$\dot{X}_{11} = (1-\alpha)\left\{(1 - \exp[-CNG_p])X_{10} + G_pX_{13} - G_{ARF}X_{11}\right\},$$

$$\dot{X}_{13} = (1-\alpha)\left\{G_{ARF}\left(CNX_{10} + X_{11}\right) - G_pX_{13}\right\},$$

$$\dot{X}_{20} = -(1-\alpha)NG_{PDGF}X_{20},$$

$$\dot{X}_{21} = (1-\alpha)NG_{PDGF}\left\{X_{20} + X_{23}C^{-1}N^{-1}\right\},$$

$$\dot{X}_{23} = -(1-\alpha)G_{PDGF}X_{23}C^{-1},$$

$$\dot{X}_{30} = -(1-\alpha)(1 - \exp[-CNG_p])X_{30},$$

$$\dot{X}_{31} = -(1-\alpha)G_pX_{31},$$

$$\dot{X}_{33} = (1-\alpha)\left\{(1 - \exp[-CNG_p])X_{30} + G_pX_{31}\right\}.$$

Here the factor $C^{-1}N^{-1}$ represents the fixation probability of the newly produced SRTA cells. Note that the rate at which the first gamma event arises in PDGF-overexpressing TA cells is given by $1 - \exp[-CNG_p]$.

Finally, let us consider the scenario in which a PDGF-overexpressing SR cell produces a TA cell clone before dying out or reaching fixation, and a TA cell from this clone acquires self-renewal. The rate at which a PDGF-overexpressing SR cell is chosen to differentiate but does not take over the SR population is given by

$$T = 2\mu\left(\alpha + \frac{1-\alpha}{2} + Nd\right)\frac{N\sum_{j=1}^{N-1}\frac{1}{j}}{\alpha + Nd}\sum_{j=1}^{N-1}\frac{N}{j}\left(\frac{1-\alpha}{\alpha + Nd}\right)\frac{j}{N}\left(\frac{N\sum_{j=1}^{N-1}\frac{1}{j}}{\alpha + Nd}\right)^{-1}.$$

$$= 2\mu\left(\alpha + \frac{1-\alpha}{2} + Nd\right)(N-1)\frac{1-\alpha}{\alpha + Nd}$$

Here $2\mu(\alpha + (1 - \alpha)/2 + Nd)$ represents the probability that a mutation arises during any time step; the parameter μ can be either μ_{ARF} or μ_{PDGF}. The expression T is denoted by T_{ARF} for cells that need to accumulate an *INK4A/ARF* mutation and by T_{PDGF} for those requiring *PDGF*-overexpression. The factor $N\sum_{j=1}^{N-1}\frac{1}{j}$ denotes the expected number of Moran (symmetric) divisions before a mutation is either fixed or lost, as derived by Rick Durrett [15]. Division by $\alpha + Nd$, the expected number of symmetric divisions per time step, leads to the expected number of time steps before fixation or loss of the mutation. We then determine the probability that in those time steps, a mutant cell is selected to asymmetrically divide and become a TA cell based on the expected time that j cells are mutated, N/j; the probability that an asymmetric division occurs while in this state, $(1 - \alpha)/(\alpha + Nd)$; and the probability of selecting a mutant cell as the asymmetrically dividing cell, j/N, summed over the number of cells j that could possibly be mutant in the SR compartment. The rate at which a gamma event occurs in such cells is given by $(1 - \exp[-NG_p])$. Here we assume that the PDGF-overexpressing SR cells will eventually be chosen to differentiate before dying out. Then the dynamics of such events that result in the cell becoming an SRTA cell is given by

$$\dot{X}_{00} = -\left\{T_{PDGF}\left(1 - \exp[-NG_p] + G_{ARF}\right) + T_{ARF}G_{PDGF}\right\}X_{00},$$

$$\dot{X}_{01} = T_{PDGF}\left\{\left(1 - \exp[-NG_p]\right)X_{00} + G_p X_{03}C^{-1}N^{-1} - G_{ARF}X_{01}C^{-1}N^{-1}\right\} - T_{ARF}G_{PDGF}X_{01}C^{-1}N^{-1},$$

$$\dot{X}_{03} = T_{PDGF}\left\{G_{ARF}\left(X_{00} + X_{01}C^{-1}N^{-1}\right) - G_p X_{03}C^{-1}N^{-1}\right\} + T_{ARF}G_{PDGF}\left\{X_{00} + X_{01}C^{-1}N^{-1}\right\},$$

$$\dot{X}_{01} = T_{PDGF}\left\{\left(1-\exp[-NG_p]\right)X_{00} + G_p X_{03}C^{-1}N^{-1} - G_{ARF}X_{01}C^{-1}N^{-1}\right\} - T_{ARF}G_{PDGF}X_{01}C^{-1}N^{-1},$$

$$\dot{X}_{03} = T_{PDGF}\left\{G_{ARF}\left(X_{00} + X_{01}C^{-1}N^{-1}\right) - G_p X_{03}C^{-1}N^{-1}\right\} + T_{ARF}G_{PDGF}\left\{X_{00} + X_{01}C^{-1}N^{-1}\right\},$$

$$\dot{X}_{10} = -T_{ARF}\left(1-\exp[-CNG_p]\right)X_{10},$$

$$\dot{X}_{11} = -T_{ARF}G_p X_{11},$$

$$\dot{X}_{13} = T_{ARF}\left\{\left(1-\exp[-CNG_p]\right)X_{10} + G_p X_{11}\right\},$$

$$\dot{X}_{20} = -T_{PDGF}\left(1-\exp[-NG_p]\right)X_{20},$$

$$\dot{X}_{21} = -T_{PDGF}G_p X_{21}C^{-1},$$

$$\dot{X}_{23} = T_{PDGF}\left\{\left(1-\exp[-NG_p]\right)X_{20} + G_p X_{21}C^{-1}\right\}.$$

In this case, the evolution of a cancer-initiating cell from a TA cell is very unlikely; it would require accumulating a mutation in the SR compartment, one such mutation differentiating before fixation or loss, gaining an additional mutation, and finally undergoing a gamma event, each of which has a low probability of occurring. Thus, we ignore such events.

Finally, we combine all separate scenarios into one system of differential equations to determine the cell of origin of PDGF-driven gliomas:

$$\dot{X}_{00} = -\begin{pmatrix} 2\left(\alpha + \frac{1-\alpha}{2} + Nd\right)\left\{\mu_{PDGF} + \mu_{ARF}\right\} + \left\{(1-\alpha)N + T_{ARF}\right\}G_{PDGF} \\ + T_{PDGF}\left(1-\exp[-NG_p] + G_{ARF}\right) \end{pmatrix} X_{00}$$

$$\begin{aligned}\dot{X}_{01} = &-\left(2\left(\alpha + \frac{1-\alpha}{2} + Nd\right)\left\{\mu_{PDGF} + \mu_{ARF}\right\} + (1+Nd)2\mu_{ARF}\right)X_{01} \\ &+ G_{PDGF}\left((1-\alpha)N\left\{X_{00} + X_{03}C^{-1}N^{-1}\right\} - T_{ARF}X_{01}C^{-1}N^{-1}\right) \\ &+ T_{PDGF}\left\{\left(1-\exp[-NG_p]\right)X_{00} + G_p X_{03}C^{-1}N^{-1} - G_{ARF}X_{01}C^{-1}N^{-1}\right\}\end{aligned}$$

$$\dot{X}_{03} = -2\left(\alpha + \frac{1-\alpha}{2} + Nd\right)\{\mu_{PDGF} + \mu_{ARF}\}X_{03} + (1+Nd)\{2\mu_{ARF}X_{01} - CN\mu_{ARF}X_{03}\}$$
$$+ T_{PDGF}\{G_{ARF}(X_{00} + X_{01}C^{-1}N^{-1}) - G_p X_{03}C^{-1}N^{-1}\}$$
$$+ G_{PDGF}(T_{ARF}\{X_{00} + X_{01}C^{-1}N^{-1}\} - (1-\alpha)X_{03}C^{-1})$$

$$\dot{X}_{10} = 2\left(\alpha + \frac{1-\alpha}{2} + Nd\right)\{\mu_{PDGF}X_{00} - \mu_{ARF}X_{10}\}$$
$$-(((1-\exp[-CNG_p])(1-\alpha-T_{ARF}) + (1-\alpha)CNG_{ARF})X_{10}$$

$$\dot{X}_{11} = 2\left(\alpha + \frac{1-\alpha}{2} + Nd\right)\{\mu_{PDGF}X_{01} - \mu_{ARF}X_{11}\}$$
$$-(1+Nd)2\mu_{ARF}X_{11} + (1-\alpha)\{(1-\exp[-CNG_p])X_{10} + G_pX_{13} - G_{ARF}X_{11}\} - T_{ARF}G_pX_{11}$$

$$\dot{X}_{13} = 2\left(\alpha + \frac{1-\alpha}{2} + Nd\right)\{\mu_{PDGF}X_{03} - \mu_{ARF}X_{13}\} + (1+Nd)\{2\mu_{ARF}X_{11} - CN\mu_{ARF}X_{13}\}$$
$$+(1-\alpha)\{G_{ARF}(CNX_{10} + X_{11}) - G_pX_{13}\} + T_{ARF}\{(1-\exp[-CNG_p])X_{10} + G_pX_{11}\}$$

$$\dot{X}_{20} = \left(\alpha + \frac{1-\alpha}{2} + Nd\right)\{2\mu_{ARF}X_{00} - (2\mu_{PDGF} + N\rho(R_{ARF})\mu_{ARF})X_{20}\}$$
$$-\{(1-\alpha)NG_{PDGF} + T_{PDGF}(1-\exp[-NG_p])\}X_{20}$$

$$\dot{X}_{21} = \left(\alpha + \frac{1-\alpha}{2} + Nd\right)\{2\mu_{ARF}X_{01} - (2\mu_{PDGF} + N\rho(R_{ARF})\mu_{ARF})X_{21}\}$$
$$-(1+Nd)2\mu_{ARF}X_{21} + (1-\alpha)NG_{PDGF}\{X_{20} + X_{23}C^{-1}N^{-1}\} - T_{PDGF}G_pX_{21}C^{-1}$$

$$\dot{X}_{23} = \left(\alpha + \frac{1-\alpha}{2} + Nd\right)\{2\mu_{ARF}X_{03} - (2\mu_{PDGF} + N\rho(R_{ARF})\mu_{ARF})X_{23}\}$$
$$+(1+Nd)\{2\mu_{ARF}X_{21} - CN\mu_{ARF}X_{23}\} - (1-\alpha)G_{PDGF}X_{23}C^{-1}$$
$$+T_{PDGF}\{(1-\exp[-NG_p])X_{20} + G_pX_{21}C^{-1}\}$$

$$\dot{X}_{30} = \left(\alpha + \frac{1-\alpha}{2} + Nd\right)\{2\mu_{PDGF}X_{20} + 2\mu_{ARF}X_{10} - CN\mu_{ARF}X_{30}\}$$
$$-(1-\alpha)CNF_{ARF}X_{33} - (1-\alpha)(1-\exp[-CNG_p])X_{30}$$

$$\dot{X}_{31} = \left(\alpha + \frac{1-\alpha}{2} + Nd\right)\{2\mu_{PDGF}X_{21} + 2\mu_{ARF}X_{11} - CN\mu_{ARF}X_{31}\}$$
$$-(1-\alpha)CNF_{ARF}X_{33} - (1+Nd)2\mu_{ARF}X_{31} - (1-\alpha)G_pX_{31}$$

$$\dot{X}_{33} = \left(\alpha + \frac{1-\alpha}{2} + Nd\right)\left\{2\mu_{PDGF}X_{23} + 2\mu_{ARF}X_{13} - CN\mu_{ARF}X_{33}\right\} - (1-\alpha)CNF_{ARF}X_{33}$$
$$+ (1+Nd)\left\{2\mu_{ARF}X_{31} - CN\mu_{ARF}X_{33}\right\} + (1-\alpha)\left\{(1 - \exp[-CNG_p])X_{30} + G_p X_{31}\right\}$$

$$\dot{X}_{40} = \left(\alpha + \frac{1-\alpha}{2} + Nd\right)N\left\{\rho(R_{ARF})\mu_{\mathrm{ARF}}X_{20} - 2\mu_{PDGF}X_{40}\right\} - (1-\alpha)NF_{PDGF}X_{40}$$

$$\dot{X}_{41} = \left(\alpha + \frac{1-\alpha}{2} + Nd\right)N\left\{\rho(R_{ARF})\mu_{\mathrm{ARF}}X_{21} - 2\mu_{PDGF}X_{41}\right\}$$
$$-(1-\alpha)NF_{PDGF}X_{41} - (1+Nd)2\mu_{ARF}X_{41}$$

$$\dot{X}_{43} = \left(\alpha + \frac{1-\alpha}{2} + Nd\right)N\left\{\rho(R_{ARF})\mu_{\mathrm{ARF}}X_{23} - 2\mu_{PDGF}X_{43}\right\}$$
$$-(1-\alpha)NF_{PDGF}X_{43} + (1+Nd)\left\{2\mu_{ARF}X_{41} - CN\mu_{ARF}X_{43}\right\}$$

$$\dot{X}_S = \left(\alpha + \frac{1-\alpha}{2} + Nd\right)N\sum_{j\in(0,1,3)}\left(C\mu_{ARF}X_{3j} + 2\mu_{PDGF}X_{4j}\right)$$

$$\dot{X}_T = (1-\alpha)N\sum_{j\in(0,1,3)}\left(CF_{ARF}X_{3j} + F_{PDGF}X_{4j}\right)$$

$$\dot{X}_R = (1+Nd)CN\mu_{ARF}\sum_{i\in(0,1,2,3,4)}X_{i3}$$

Here we have

$$F_{ARF} = \frac{1}{2}(1+2d)\mu_{ARF}\sum_{k=0}^{z}\sum_{l=k}^{z+\beta_{ARF}}2^l(\gamma - l\gamma_{step})$$

$$F_{PDGF} = \frac{1}{2}(1+2d)\mu_{PDGF}\sum_{k=0}^{z+\beta_{ARF}}\sum_{l=k}^{z+\beta_{ARF}}2^l(\gamma - l\gamma_{step})$$

$$G_p = \sum_{k=0}^{z}2^k\left(\gamma - k\gamma_{step}\right)$$

$$G_{ARF} = \frac{1}{2}(1+2d)\mu_{ARF}\sum_{k=0}^{z}\sum_{l=k}^{z}2^l\left(\gamma - l\gamma_{step}\right)$$

$$G_{PDGF} = \frac{1}{2}(1+2d)\mu_{PDGF}\sum_{k=0}^{z}\sum_{l=k}^{z}2^l\left(\gamma - l\gamma_{step}\right)$$

$$T_{ARF} = 2\mu_{ARF}\left(\alpha + \frac{1-\alpha}{2} + Nd\right)(N-1)\frac{1-\alpha}{\alpha + Nd}$$

$$T_{PDGF} = 2\mu_{PDGF}\left(\alpha + \frac{1-\alpha}{2} + Nd\right)(N-1)\frac{1-\alpha}{\alpha + Nd}$$

We determined the equations for the NF1-driven case in a manner similar to the PDGF-driven case. The basic system remains the same; all that changes is the effect of each mutation. We consider per allele per division mutation rates of μ_{NF1} and μ_{TP53} for *NF1* loss and *TP53* mutation. Alteration of *TP53* results in a relative fitness of R_{TP53} in SR cells and an additional β_{TP53} cell divisions in TA cells. Also, all cells with the mutant *TP53* allele no longer undergo accidental cell death. Bi-allelic inactivation of *NF1* results in a decreased growth rate of R_{NF1} in SR cells and no additional cell divisions in TA cells, unless combined with other alterations. All initial conditions remain the same: all cells are unmutated, and the SRTA compartment does not exist if there is no appreciable gamma effect associated with NF1 loss (see main text for discussion of alternative assumptions).

We denote by Y_{ij} the probability that the SR and SRTA cell populations are in states i and j, and by X_S, X_T, and X_R the probabilities that a cancer-initiating clone arises from an SR, TA, and SRTA cell, respectively. We enumerate the states in Table S2. Let us first consider only the dynamics within the SR cell population, given by

$$\dot{Y}_{0j} = -2\left(\alpha + \frac{1-\alpha}{2} + Nd\right)\left\{N\rho(R_{TP53})\mu_{TP53} + \mu_{NF1}\right\}Y_{0j},$$

$$\dot{Y}_{1j} = 2\left(\alpha + \frac{1-\alpha}{2} + Nd\right)N\rho(R_{TP53})\mu_{TP53}Y_{0j} - 2\left(\alpha + \frac{1-\alpha}{2}\right)\mu_{NF1}Y_{1j},$$

$$\dot{Y}_{2j} = \left(\alpha + \frac{1-\alpha}{2} + Nd\right)\left\{2\mu_{NF1}Y_{0j} - N\left(2\rho(R_{TP53})\mu_{TP53} + \rho(R_{NF1,wt})\mu_{NF1}\right)Y_{2j}\right\}, \text{for all } j \in (0,1,3)$$

$$\dot{Y}_{3j} = \left(\alpha + \frac{1-\alpha}{2}\right)\left\{2\mu_{NF1}Y_{1j} - N\mu_{NF1}Y_{3j}\right\} + 2\left(\alpha + \frac{1-\alpha}{2} + Nd\right)N\rho(R_{TP53})\mu_{TP53}Y_{2j},$$

$$\dot{Y}_{4j} = \left(\alpha + \frac{1-\alpha}{2} + Nd\right) N\left\{\rho\left(R_{NF1,wt}\right)\mu_{NF1} Y_{2j} - 2\mu_{TP53} Y_{4j}\right\},$$

$$\dot{Y}_S = \left(\alpha + \frac{1-\alpha}{2}\right) N\mu_{NF1} \sum_j Y_{3j} + \left(\alpha + \frac{1-\alpha}{2} + Nd\right) 2N\mu_{TP53} \sum_j Y_{4j}.$$

The factors $\rho(R_{NF1,wt})$ and $\rho(R_{TP53})$ are defined in the same manner as $\rho(R_{ARF})$.

Next, we consider the SRTA compartment alone. The dynamics of this compartment are given by

$$\dot{Y}_{i1} = -2\mu_{NF1} Y_{i1},$$

$$\dot{Y}_{i3} = 2\mu_{NF1} Y_{i1} - N\mu_{NF1} Y_{i3}, \qquad \text{for all } i \in (0,1,2,3,4)$$

$$\dot{Y}_R = N\mu_{NF1} \sum_i Y_{i3}.$$

In order for TA cells to either become cancerous or transfer to the SRTA compartment, some event that confers self-renewal must occur; this event arises at rate γ. In the NF1 case, there is no possibility for such event. Thus, the following equations all result in a change of zero:

$$F_{NF1} = \frac{1}{2}\mu_{NF1} \sum_{k=0}^{z} \sum_{l=k}^{z} 2^l (\gamma - l\gamma_{step}) = 0$$

$$F_{TP53} = \frac{1}{2}(1+2d)\mu_{TP53} \sum_{k=0}^{z} \sum_{l=k}^{z+\beta_{TP53}} 2^l (\gamma - l\gamma_{step}) = 0$$

$$G_t = \sum_{k=0}^{z} 2^k \left(\gamma - k\gamma_{step}\right) = 0$$

$$G_{NF1} = \frac{1}{2}(1+2d)\mu_{NF1} \sum_{k=0}^{z+\beta_{TP53}} \sum_{l=k}^{z+\beta_{TP53}} 2^l \left(\gamma - l\gamma_{step}\right) = 0$$

$$G_{TP53} = \frac{1}{2}(1+2d)\mu_{TP53} \sum_{k=0}^{z} \sum_{l=k}^{z} 2^l \left(\gamma - l\gamma_{step}\right) = 0$$

Since all F equations are zero in the NF1-driven case, all equations for TA cell cancer initiation are zero. Similarly, all G equations are zero, so there is no transfer of cells from the TA compartment to the SRTA compartment.

Let us now investigate the parameter dependence of the probabilities of cancer initiation from the different cell populations: from SR cells (shown in red and denoted by X_S and Y_S), TA cells (shown in blue and denoted by X_T and Y_T), and SRTA cells (shown in green and denoted by X_R and Y_R) (Figs. S1-S4). The analytical approximations of all three probabilities demonstrate a good fit with the results of the exact stochastic computer simulations (Fig. S1 and S2). Note the divergence in results between the NF1- and PDGF-driven cases. In the NF1-driven case, the only cell type capable of initiating cancer is the SR cell type, regardless of the parameters used (Fig. S2). This is a result of the need for some form of self-renewal capability in order to maintain a cancerous population. However, in the PDGF-driven case, SRTA cells almost always have the highest probability of cancer initiation. Only in the case where the probability of regaining self-renewal capabilities is sufficiently low (below ~2-5×10^{-5}) does any other cell type have a higher probability of cancer initiation. Therefore, in the PDGF-driven case, SR cells are most likely to initiate gliomagenesis. Overall, the probability of cancer initiation from TA cells, X_T, is almost always lowest. In only a few cases, namely when z or β_{ARF} is large, is X_T not the lowest probability. However, in all those cases, X_R is always larger.

Since the probability of cancer initiating from non-SR cells in the NF1 case is zero, due to the assumption of no gamma effect in this case, we consider the effects of each parameter in the PDGF case only. The rate of symmetric SR cell division, α, has little effect on the probabilities (Fig. S3*A*). Also, X_T increases with the number of additional cell divisions that *INK4A/ARF*$^{-/-}$ TA cells can undergo, β_{ARF}, while the other probabilities show little dependence on that parameter (Fig. S3*B*). The probability of cancer initiation from the self-renewing TA cell population increases with the expansion factor resulting from PDGF overexpression, C (Fig. S3*C*). The probability of an individual cell dying per time step, d, results in an increase in the probabilities of cancer initiation from all three cell types, although not equally. However, even at very high cell death rates (1 in 10 cells dying every time step), the probability of cancer initiation from SRTA cells is still considerably larger than the probability of cancer initiation from any other

cell type (Fig. S3*D*). The rate at which PDGF-overexpressing TA cells gain self-renewal capabilities, γ, and rate of decrease of that probability with increasing differentiation, γ_{step}, enhances X_R and, to a lesser extent, X_T as γ increases and γ_{step} decreases (Fig. S3*E*, *F*, *G*). Higher mutation rates increase the probabilities of cancer initiation from all three cell types (Fig. S3*I-K*). Interestingly, when the number of SR cells (*N*) is large, the total probability of cancer initiation is small because both the time until fixation and the risk of extinction of mutated SR cells increase (Fig. S3*H*). The three probabilities are not significantly influenced by the relative fitness of *INK4A/ARF*$^{-/-}$ SR cells, R_{ARF} (Fig. S3*L*). The number of cell divisions TA cells can undergo, *z*, increases primarily X_R but also to a lesser extent X_T since there is an increased chance for acquiring mutations and self-renewal capabilities when *z* is large (Fig. S3*M*). These results hold true even when we vary the ratio of symmetric to asymmetric differentiation divisions, λ, (Fig. S4).

The predictions of our models are robust with regard to changes in parameters (Fig. 5*B*). However, it is possible that other combinations of genetic alterations within the same subtype may result in gliomagenesis. Indeed, many mutations in the same pathway may be functionally equivalent [16,17,18,19,20,21]. Any such alterations would be expected to result in a similar phenotype; thus, they would act functionally as if one of the genes already included in the model were altered. In the model, this would be equivalent to increasing the mutation rate by a factor equal to the number of potential replacement mutations. As we have shown (Fig. S3*I-K*), increases in mutation rate even by orders of magnitude do not significantly change our results; therefore, our conclusions will hold when mutations with similar phenotype to already included alterations are added to the model. Nonetheless, should further mutational effects be discovered, new extensions to this model would need to be developed.

Since the mathematical model described here represents only one possible approach to modeling the evolutionary dynamics leading to glioma formation, we also investigated the robustness of our conclusions to the model assumptions. If there are multiple independent TA cell populations originating from each SR population, then the identity of the cell of origin does not change appreciably (data not shown). If the SVZ is not subdivided into independent cell clusters but consists of a single well-mixed population of stem cells, then the conclusions of the model are

robust as well (Fig. S3*H*). If the PDGF-driven SRTA cell population divides asymmetrically as well as symmetrically, thus producing its own non-self-renewing TA cells, it is possible that the final event before cancer initiation is a gamma event in these cells. However, these cells would still be TA cells that have acquired self-renewal properties. Recent modeling work has shown an inhibitory effect that large numbers of differentiated tumor cells may have on tumor mass growth, due to spatial competition [22]. Thus, the large pool of terminally differentiated cells may have an effect on the dynamics of tumor development. However, since we consider the initiation of a tumor, before large-scale aberrant growth occurs, we expect little spatial competition. At this point, we assume the terminally differentiated cells in the model are more similar to normal differentiated cells and thus die out normally. Given the lack of knowledge surrounding the rate at which mutations not linked to cell division arise, we neglect the terminally differentiated cell pool as a viable alternative cell of origin. We therefore conclude that even if the structure of the mathematical model varies, the most likely cell of origin of PDGF-driven gliomas is still a transit-amplifying cell that has evolved the property of undergoing symmetric self-renewing divisions. Similarly, the model describing NF1-driven gliomas is very robust with regard to the modeling assumptions. The only change that would allow for a TA cell to serve as the cell of origin of this subtype of glioma would be the consideration of an (epi)genetic event that allows such cells to undergo self-renewing divisions. In the presence of such a possibility, TA cells may be the most likely cells of origin. However, since there is no evidence for such an alteration arising in NF1-driven cases, we disregard this possibility in our model.

Supplemental References

1. Bondiau PY, Clatz O, Sermesant M, Marcy PY, Delingette H, et al. (2008) Biocomputing: numerical simulation of glioblastoma growth using diffusion tensor imaging. Phys Med Biol 53: 879-893.
2. Burgess PK, Kulesa PM, Murray JD, Alvord EC, Jr. (1997) The interaction of growth rates and diffusion coefficients in a three-dimensional mathematical model of gliomas. J Neuropathol Exp Neurol 56: 704-713.
3. Deroulers C, Aubert M, Badoual M, Grammaticos B (2009) Modeling tumor cell migration: From microscopic to macroscopic models. Phys Rev E Stat Nonlin Soft Matter Phys 79: 031917.

4. Eikenberry SE, Sankar T, Preul MC, Kostelich EJ, Thalhauser CJ, et al. (2009) Virtual glioblastoma: growth, migration and treatment in a three-dimensional mathematical model. Cell Prolif 42: 511-528.
5. Frieboes HB, Lowengrub JS, Wise S, Zheng X, Macklin P, et al. (2007) Computer simulation of glioma growth and morphology. Neuroimage 37 Suppl 1: S59-70.
6. Hogea C, Davatzikos C, Biros G (2008) An image-driven parameter estimation problem for a reaction-diffusion glioma growth model with mass effects. J Math Biol 56: 793-825.
7. Newman WI, Lazareff JA (2003) A mathematical model for self-limiting brain tumors. J Theor Biol 222: 361-371.
8. Sander LM, Deisboeck TS (2002) Growth patterns of microscopic brain tumors. Phys Rev E Stat Nonlin Soft Matter Phys 66: 051901.
9. Swanson KR, Bridge C, Murray JD, Alvord EC, Jr. (2003) Virtual and real brain tumors: using mathematical modeling to quantify glioma growth and invasion. J Neurol Sci 216: 1-10.
10. Szeto MD, Chakraborty G, Hadley J, Rockne R, Muzi M, et al. (2009) Quantitative metrics of net proliferation and invasion link biological aggressiveness assessed by MRI with hypoxia assessed by FMISO-PET in newly diagnosed glioblastomas. Cancer Res 69: 4502-4509.
11. Tanaka ML, Debinski W, Puri IK (2009) Hybrid mathematical model of glioma progression. Cell Prolif 42: 637-646.
12. Wise SM, Lowengrub JS, Frieboes HB, Cristini V (2008) Three-dimensional multispecies nonlinear tumor growth--I Model and numerical method. J Theor Biol 253: 524-543.
13. Mirzadeh Z, Merkle FT, Soriano-Navarro M, Garcia-Verdugo JM, Alvarez-Buylla A (2008) Neural stem cells confer unique pinwheel architecture to the ventricular surface in neurogenic regions of the adult brain. Cell Stem Cell 3: 265-278.
14. Haeno H, Levine RL, Gilliland DG, Michor F (2009) A progenitor cell origin of myeloid malignancies. Proc Natl Acad Sci U S A 106: 16616-16621.
15. Durrett R (2008) Moran model. In: Gani JH, C. C.; Jagers, P.; Jurtz, T. G., editor. Probability Models for DNA Sequence Evolution. New York: Springer Science+Business Media, LLC. pp. 46-50.
16. Ueki K, Ono Y, Henson JW, Efird JT, von Deimling A, et al. (1996) CDKN2/p16 or RB alterations occur in the majority of glioblastomas and are inversely correlated. Cancer Res 56: 150-153.
17. Cerami E, Demir E, Schultz N, Taylor BS, Sander C (2010) Automated network analysis identifies core pathways in glioblastoma. PLoS One 5: e8918.
18. Biernat W, Tohma Y, Yonekawa Y, Kleihues P, Ohgaki H (1997) Alterations of cell cycle regulatory genes in primary (de novo) and secondary glioblastomas. Acta Neuropathol 94: 303-309.
19. Nishikawa R, Furnari FB, Lin H, Arap W, Berger MS, et al. (1995) Loss of P16INK4 expression is frequent in high grade gliomas. Cancer Res 55: 1941-1945.
20. Costello JF, Plass C, Arap W, Chapman VM, Held WA, et al. (1997) Cyclin-dependent kinase 6 (CDK6) amplification in human gliomas identified using two-dimensional separation of genomic DNA. Cancer Res 57: 1250-1254.
21. Burns KL, Ueki K, Jhung SL, Koh J, Louis DN (1998) Molecular genetic correlates of p16, cdk4, and pRb immunohistochemistry in glioblastomas. J Neuropathol Exp Neurol 57: 122-130.

22. Enderling H, Hahnfeldt P (2011) Cancer stem cells in solid tumors: Is 'evading apoptosis' a hallmark of cancer? Prog Biophys Mol Biol.

Supplemental Tables

Table S1. Definition of mutational states of the SR and SRTA cell populations for PDGF-driven glioma. Each state denotes a homogeneous population of SR or SRTA cells harboring the respective genetic alterations.

State 00	The SR population consists of only unmutated cells and there is no SRTA population
State 10	The SR population consists of only PDGF-overexpressing cells and there is no SRTA population
State 20	The SR population consists of only *INK4A/ARF*$^{+/-}$ cells and there is no SRTA population
State 30	The SR population consists of only *INK4A/ARF*$^{+/-}$ cells that overexpress PDGF and there is no SRTA population
State 40	The SR population consists of only *INK4A/ARF*$^{-/-}$ cells and there is no SRTA population
State 01	The SR population consists of only unmutated cells and the SRTA population consists of only PDGF-overexpressing cells
State 11	Both the SR and the SRTA populations consist of only PDGF-overexpressing cells
State 21	The SR population consists of only *INK4A/ARF*$^{+/-}$ cells and the SRTA population consists of only PDGF-overexpressing cells
State 31	The SR population consists of only *INK4A/ARF*$^{+/-}$ cells that overexpress PDGF and the SRTA population consists of only PDGF-overexpressing cells
State 41	The SR population consists of only *INK4A/ARF*$^{-/-}$ cells and the SRTA population consists of only PDGF-overexpressing cells

State 03	The SR population consists of only unmutated cells and the SRTA population consists of only *INK4A/ARF*$^{+/-}$ cells that overexpress PDGF
State 13	The SR population consists of only PDGF-overexpressing cells and the SRTA population consists of *INK4A/ARF*$^{+/-}$ cells that overexpress PDGF
State 23	The SR population consists of only *INK4A/ARF*$^{+/-}$ cells and the SRTA population consists of only *INK4A/ARF*$^{+/-}$ cells that overexpress PDGF
State 33	Both the SR and SRTA populations consist of only *INK4A/ARF*$^{+/-}$ cells that overexpress PDGF
State 43	The SR population consists of only *INK4A/ARF*$^{-/-}$ cells and the SRTA population consists of only *INK4A/ARF*$^{+/-}$ cells that oveerxpress PDGF
State S	There exists at least one *INK4A/ARF*$^{-/-}$ cell that overexpresses PDGF in the SR population, leading to cancer initiation
State R	There exists at least one *INK4A/ARF*$^{-/-}$ cell that overexpresses PDGF in the SRTA population, leading to cancer initiation
State T	There exists at least one *INK4A/ARF*$^{-/-}$ cell that overexpresses PDGF in the TA population that successfully gained self-renewal before terminally differentiating, thus initiating cancer

Table S2. Definition of mutational states of the SR and SRTA cell populations for NF1-driven glioma. Each state denotes a homogeneous population of SR or SRTA cells harboring the respective genetic alterations. Note that in this case, we assume that there is no appreciable gamma effect associated with NF1 loss; see the main text for discussion of alternative assumptions.

State 00	The SR population consists of only unmutated cells and there is no SRTA population
State 10	The SR population consists of only *TP53*-dominant negative cells and there is no SRTA population
State 20	The SR population consists of only *NF1*$^{+/-}$ cells and there is no SRTA population

State 30	The SR population consists of only *NF1*$^{+/-}$ *TP53*-dominant negative cells and there is no SRTA population
State 40	The SR population consists of only *NF1*$^{-/-}$ cells and there is no SRTA population
State 01	The SR population consists of only unmutated cells and the SRTA population consists of only *TP53*-dominant negative cells
State 11	Both the SR and the SRTA populations consist of only *TP53*-dominant negative cells
State 21	The SR population consists of only *NF1*$^{+/-}$ cells and the SRTA population consists of only *TP53*-dominant negative cells
State 31	The SR population consists of only *NF1*$^{+/-}$ *TP53*-dominant negative cells and the SRTA population consists of only *TP53*-dominant negative cells
State 41	The SR population consists of only *NF1*$^{-/-}$ cells and the SRTA population consists of only *TP53*-dominant negative cells
State 03	The SR population consists of only unmutated cells and the SRTA population consists of only *NF1*$^{+/-}$ *TP53*-dominant negative cells
State 13	The SR population consists of only *TP53*-dominant negative cells and the SRTA population consists of *NF1*$^{+/-}$ *TP53*-dominant negative cells
State 23	The SR population consists of only *NF1*$^{+/-}$ cells and the SRTA population consists of only *NF1*$^{+/-}$ *TP53*-dominant negative cells
State 33	Both the SR and SRTA populations consist of only *NF1*$^{+/-}$ *TP53*-dominant negative cells
State 43	The SR population consists of only *NF1*$^{-/-}$ cells and the SRTA population consists of only *NF1*$^{+/-}$ *TP53*-dominant negative cells
State S	There exists at least one *NF1*$^{-/-}$ *TP53*-dominant negative cell in the SR population, leading to cancer initiation
State R	There exists at least one *NF1*$^{-/-}$ *TP53*-dominant negative cell in the SRTA population, leading to cancer initiation
State T	There exists at least one *NF1*$^{-/-}$ *TP53*-dominant negative cell in the TA population that successfully gained self-renewal before terminally differentiating, thus initiating cancer

Supplemental Figures

Figure S1. Fit of the analytical approximation to simulation results for PDGF-driven gliomas. We investigate the fit of the analytical approximation derived for the PDGF-driven case with the output of exact stochastic computer simulations while varying each parameter and keeping the other values constant. Dots represent the results of the exact stochastic computer simulations and curves represent the results of the analytical approximation. The black curve shows the total probability of cancer initiation, the red curve the probability of cancer initiation from self-renewing (SR) cells, the blue curve the probability of cancer initiation from transit-amplifying (TA) cells, and the green curve the probability of cancer initiation from self-renewing transit-amplifying (SRTA) cells. The standard parameter values are α = 0.2 (probability of a symmetric SR cell division); β_{ARF} = 1 (additional number of cell divisions of *INK4A/ARF*$^{-/-}$ TA cells); C = 3 (expansion factor due to PDGF overexpression); d = 0.005 (per cell per division accidental death rate); γ = 0.005 (rate of acquisition of self-renewal in the most undifferentiated PDGF-overexpressing TA cells); γ_{steo} = 0.0005 (reduction factor of γ with each cell division); $\mu_{ARF} = \mu_{PDGF} = 10^{-4}$ (mutation rate per allele); N = 5 (number of SR cells); R_{ARF} = 1.1 (relative fitness value (i.e. growth rate) of *INK4A/ARF*$^{-/-}$ SR cells); z = 3 (number of TA cell divisions);, and t = 4000 (time).

Figure S2. Fit of the analytical approximation to simulation results for NF1-driven gliomas. We investigate the fit of the analytical approximation in the PDGF-driven case with the output of exact stochastic computer simulations while varying each parameter and keeping the other values constant. Dots represent the results of the exact stochastic computer simulations and curves represent the results of the analytical approximation. The red curve shows the probability of cancer initiation from self-renewing (SR) cells; all other cell types are zero and so are not displayed. Note that this latter effect arises since we assume that there is no appreciable gamma effect associated with NF1 loss; see the main text for discussion of alternative assumptions. The standard parameter values are α = 0.2 (probability of a symmetric SR cell division); $\beta_{NF1} = \beta_{TP53}$ = 1 (additional number of cell divisions of *NF1*$^{-/-}$ and *TP53*-dominant negative TA cells); d = 0.005 (per cell per division accidental death rate); $\mu_{NF1} = \mu_{TP53} = 10^{-4}$ (mutation rate per

allele); $N = 5$ (number of SR cells); $R_{NF1,wt} = 0.2$ (relative fitness value (i.e. growth rate) of *NF1*$^{-/-}$ mutant SR cells without *TP53* or *INK4A/ARF* mutations); $R_{NF1,mut} = 1.1$ (relative fitness value (i.e. growth rate) of *NF1*$^{-/-}$ mutant SR cells with *TP53* or *INK4A/ARF* mutations); $R_{TP53} = 1.1$ (relative fitness value (i.e. growth rate) of *TP53* dominant negative SR cells); $z = 3$ (number of TA cell divisions), and $t = 4000$ (time).

Figure S3. Parameter dependence of the probabilities of cancer initiation for PDGF-driven gliomas using mutation rates of human cells. We investigate the parameter dependence of the probabilities of cancer initiation using the differential equation systems in the PDGF-driven case by varying each parameter while keeping the other values constant. The red curve shows the probability of cancer initiation from self-renewing (SR) cells, the blue curve the probability of cancer initiation from transit-amplifying (TA) cells, and the green curve the probability of cancer initiation from self-renewing transit-amplifying (SRTA) cells. Parameters are kept the same as when fitting the approximation to simulation (Fig. S1), but the default mutation rate is decreased to $\mu_{ARF} = \mu_{PDGF} = 10^{-7}$.

Figure S4. Parameter dependence of the probabilities of cancer initiation for PDGF-driven gliomas when symmetric differentiation is introduced. We investigate the parameter dependence of the probabilities of cancer initiation in the NF1-driven case by varying each parameter while keeping the other values constant. The red curve shows the probability of cancer initiation from self-renewing (SR) cells, the blue curve the probability of cancer initiation from transit-amplifying (TA) cells, and the green curve the probability of cancer initiation from self-renewing transit-amplifying (SRTA) cells. Parameters are kept the same as when fitting the approximation to simulation (Fig. S1), but only asymmetric differentiation (solid lines), half symmetric differentiation steps and half asymmetric differentiation steps (dashed lines), and all symmetric differentiation steps (dotted lines) are displayed. These results are derived from the exact stochastic computer simulations.

Figure S5. Effect of therapeutic interventions that increase the death rates in SR, TA, and SRTA cells, and all combinations thereof on PDGF-driven gliomagenesis. We investigate the effect of treatments that selectively increase cell death in **A)** SR cells, **B)** TA cells, **C)** SRTA

cells, **D)** SR and TA cells, **E)** SR and SRTA cells, and **F)** TA and SRTA cells. The red curve shows the probability of cancer initiation from self-renewing (SR) cells, the blue curve the probability of cancer initiation from transit-amplifying (TA) cells, and the green curve the probability of cancer initiation from self-renewing transit-amplifying (SRTA) cells. All parameters are kept the same as in Figure S1 except for death rates.

Fig. S1

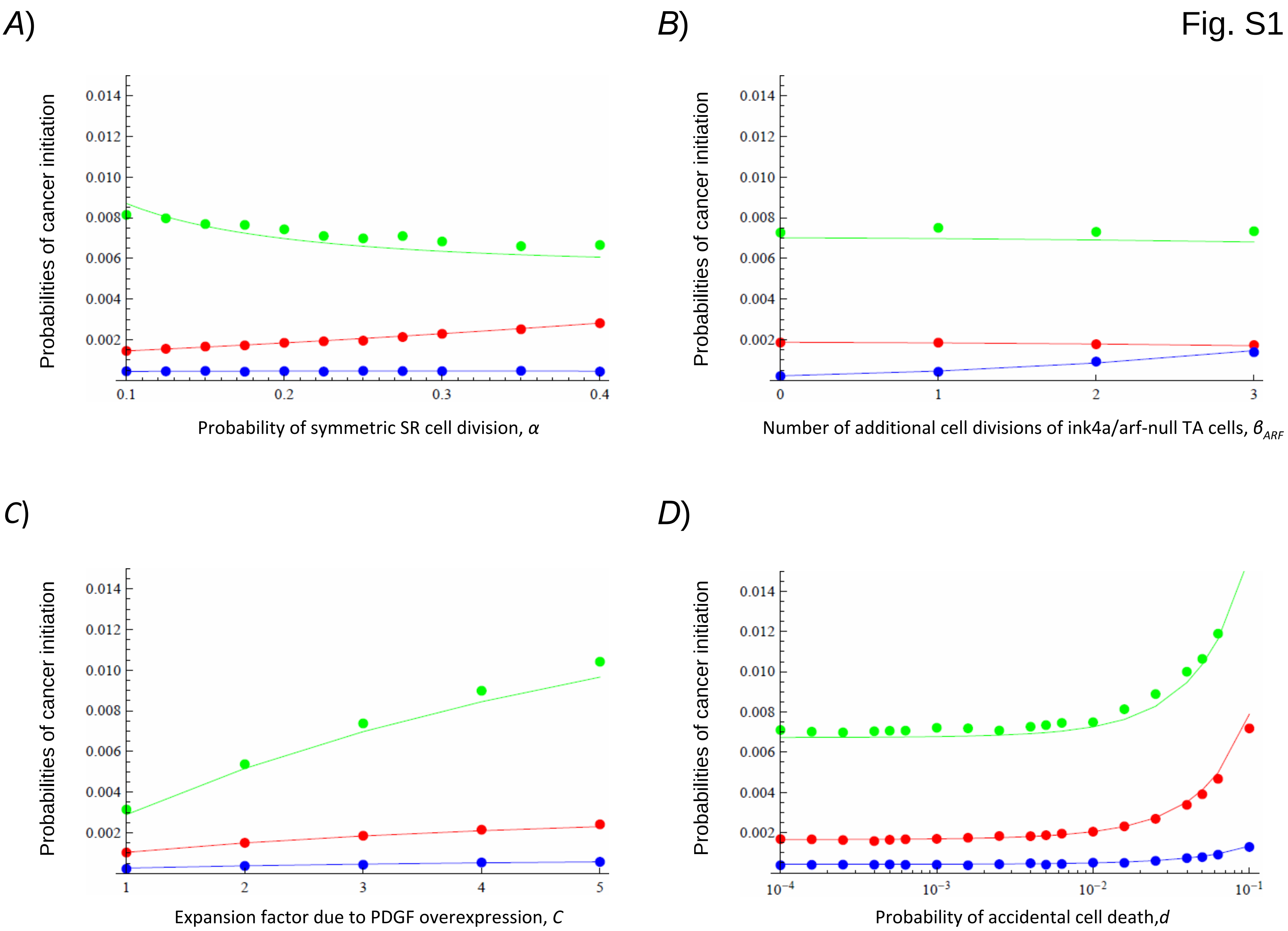
A)
Probabilities of cancer initiation
0.014
0.012
0.010
0.008
0.006
0.004
0.002
0.1
0.2
0.3
0.4
Probability of symmetric SR cell division, α
B)
Probabilities of cancer initiation
0.014
0.012
0.010
0.008
0.006
0.004
0.002
0
1
2
3
Number of additional cell divisions of ink4a/arf-null TA cells, β_{ARF}
C)
Probabilities of cancer initiation
0.014
0.012
0.010
0.008
0.006
0.004
0.002
1
2
3
4
5
Expansion factor due to PDGF overexpression, C
D)
Probabilities of cancer initiation
0.014
0.012
0.010
0.008
0.006
0.004
0.002
10^{-4}
10^{-3}
10^{-2}
10^{-1}
Probability of accidental cell death,d

Fig. S1

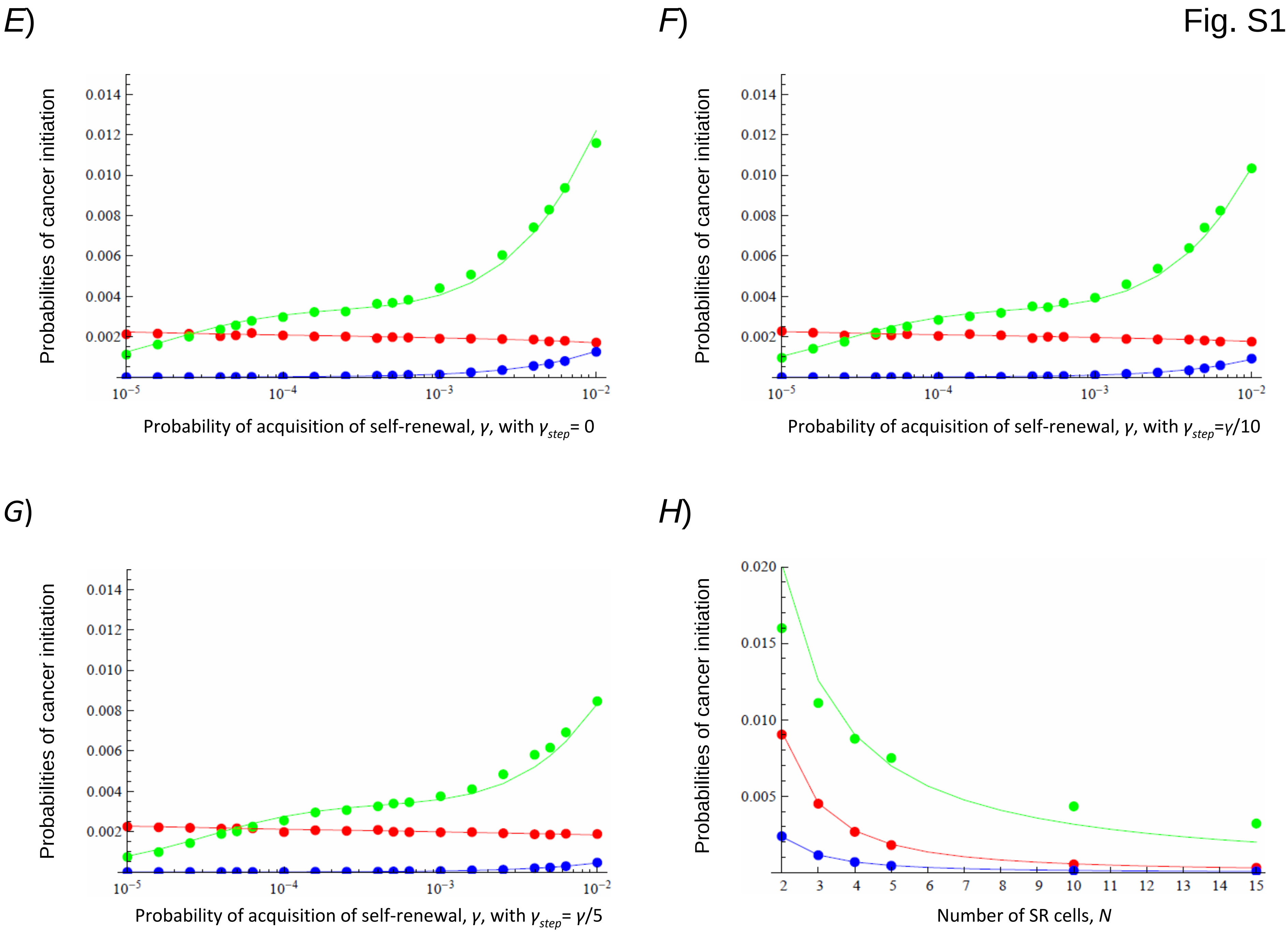
E)
F)
G)
H)
Probabilities of cancer initiation
0.014
0.012
0.010
0.008
0.006
0.004
0.002
10^{-5}
10^{-4}
10^{-3}
10^{-2}
Probability of acquisition of self-renewal, γ, with γ_{step}= 0
Probability of acquisition of self-renewal, γ, with γ_{step}=γ/10
Probability of acquisition of self-renewal, γ, with γ_{step}= γ/5
0.020
0.015
0.010
0.005
2 3 4 5 6 7 8 9 10 11 12 13 14 15
Number of SR cells, N

Fig. S1

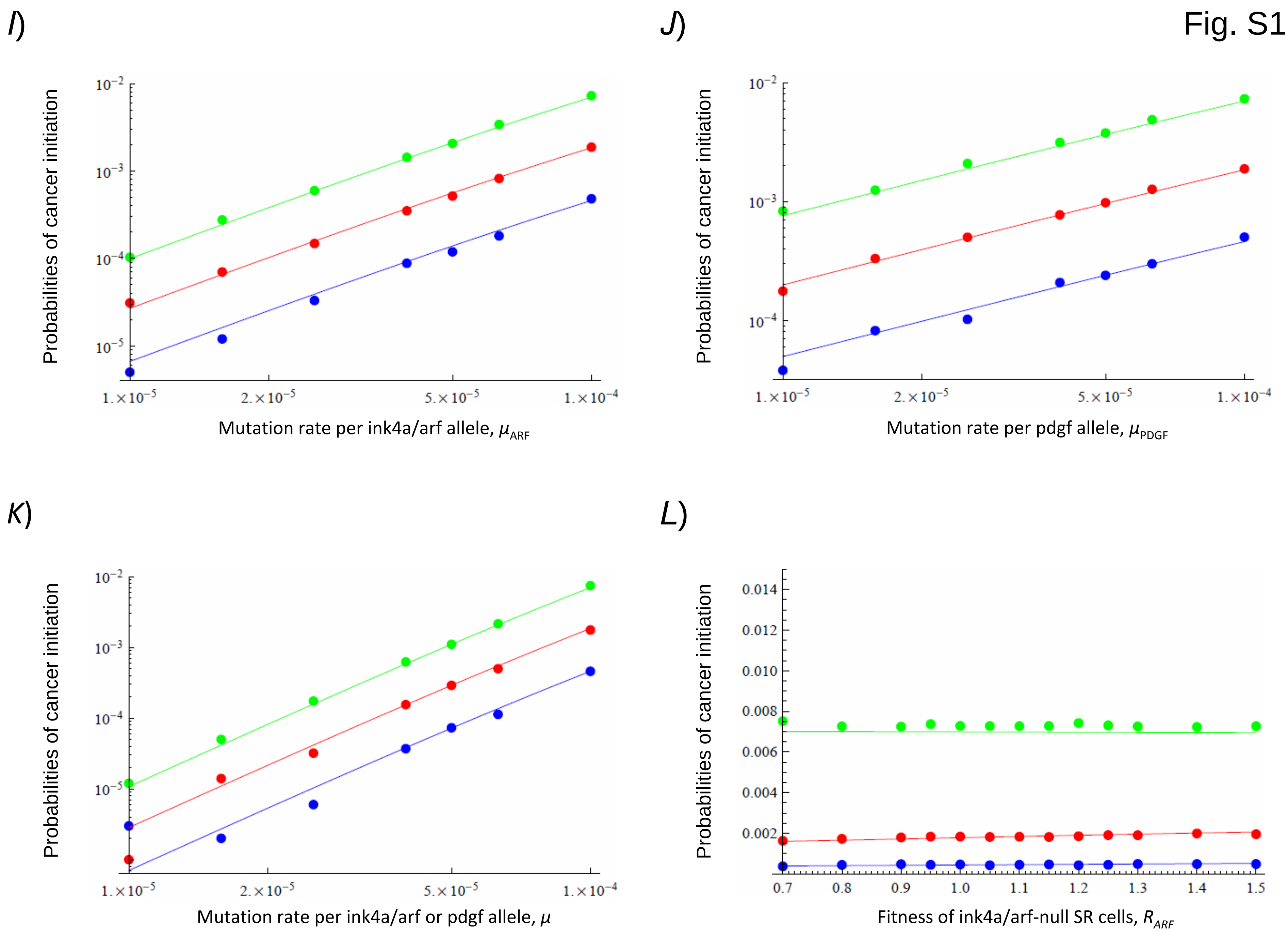
I)
J)
K)
L)
Probabilities of cancer initiation
Mutation rate per ink4a/arf allele, μ_{ARF}
Mutation rate per pdgf allele, μ_{PDGF}
Mutation rate per ink4a/arf or pdgf allele, μ
Fitness of ink4a/arf-null SR cells, R_{ARF}
10^{-2}
10^{-3}
10^{-4}
10^{-5}
$1.\times10^{-5}$
$2.\times10^{-5}$
$5.\times10^{-5}$
$1.\times10^{-4}$
0.014
0.012
0.010
0.008
0.006
0.004
0.002
0.7
0.8
0.9
1.0
1.1
1.2
1.3
1.4
1.5

M)

Fig. S1

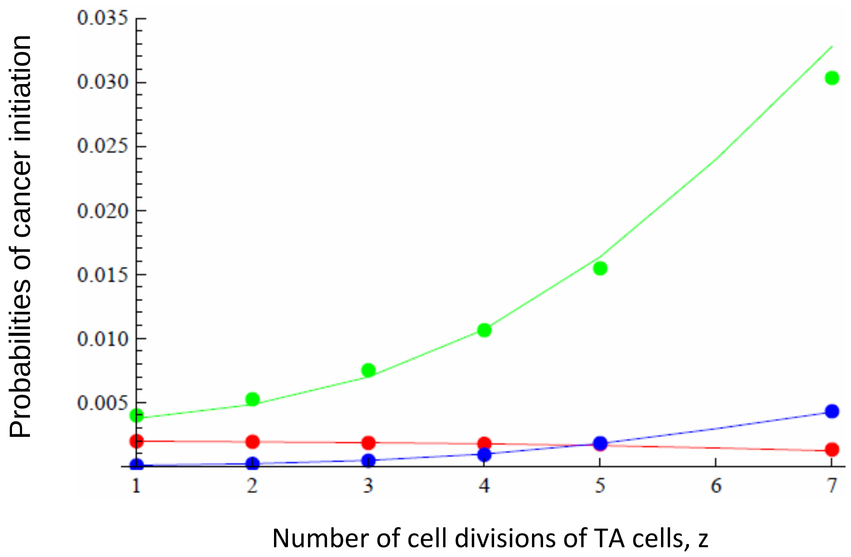

Probabilities of cancer initiation
0.035
0.030
0.025
0.020
0.015
0.010
0.005
1
2
3
4
5
6
7
Number of cell divisions of TA cells, z

Fig. S2

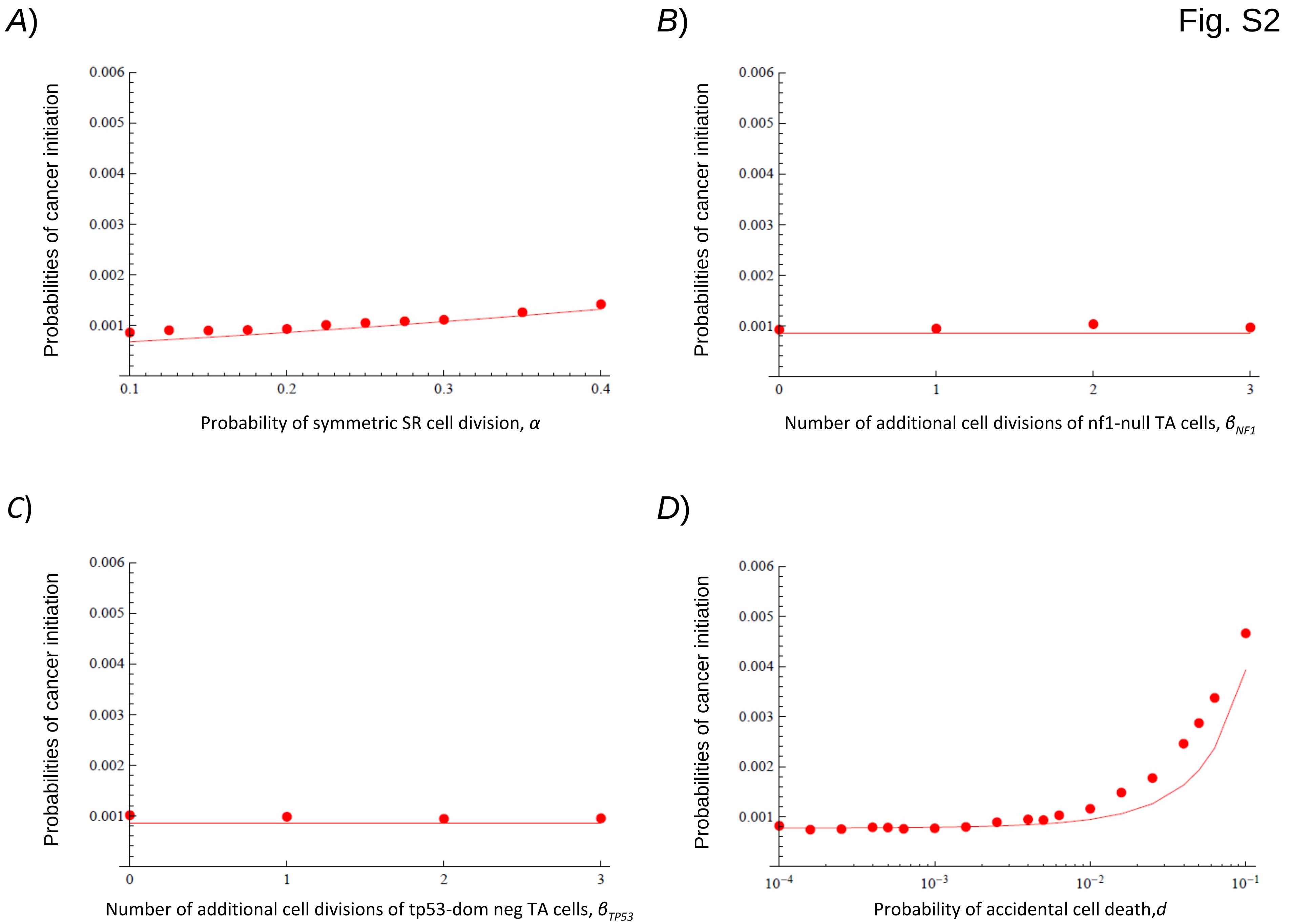
A)
Probabilities of cancer initiation
0.006
0.005
0.004
0.003
0.002
0.001
0.1
0.2
0.3
0.4
Probability of symmetric SR cell division, α
B)
Probabilities of cancer initiation
0.006
0.005
0.004
0.003
0.002
0.001
0
1
2
3
Number of additional cell divisions of nf1-null TA cells, β_NF1
C)
Probabilities of cancer initiation
0.006
0.005
0.004
0.003
0.002
0.001
0
1
2
3
Number of additional cell divisions of tp53-dom neg TA cells, β_TP53
D)
Probabilities of cancer initiation
0.006
0.005
0.004
0.003
0.002
0.001
10^-4
10^-3
10^-2
10^-1
Probability of accidental cell death,d

Fig. S2

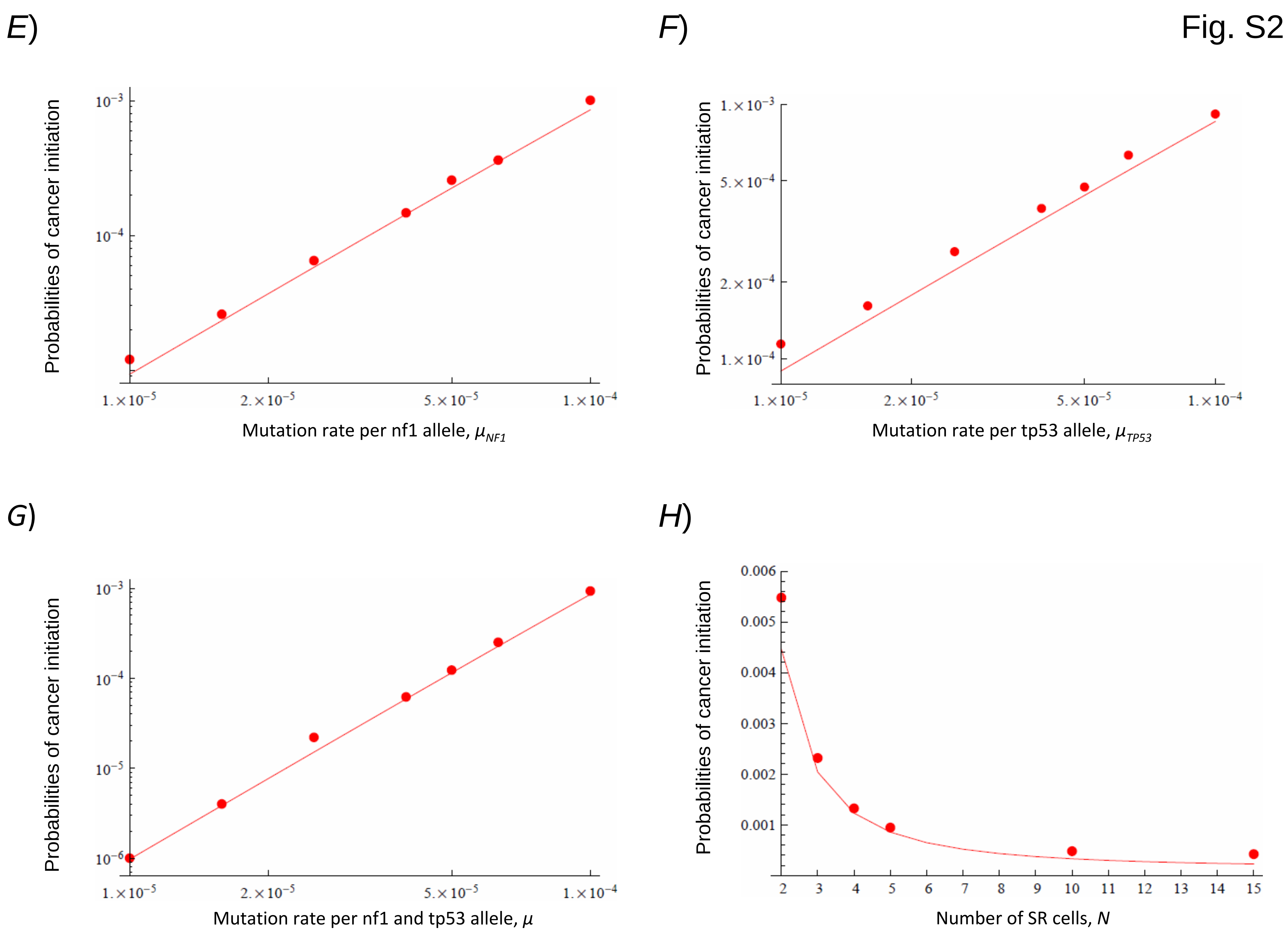

E)
Probabilities of cancer initiation
10^-3
10^-4
1.×10^-5
2.×10^-5
5.×10^-5
1.×10^-4
Mutation rate per nf1 allele, μ_NF1
F)
Probabilities of cancer initiation
1.×10^-3
5.×10^-4
2.×10^-4
1.×10^-4
1.×10^-5
2.×10^-5
5.×10^-5
1.×10^-4
Mutation rate per tp53 allele, μ_TP53
G)
Probabilities of cancer initiation
10^-3
10^-4
10^-5
10^-6
1.×10^-5
2.×10^-5
5.×10^-5
1.×10^-4
Mutation rate per nf1 and tp53 allele, μ
H)
Probabilities of cancer initiation
0.006
0.005
0.004
0.003
0.002
0.001
2
3
4
5
6
7
8
9
10
11
12
13
14
15
Number of SR cells, N

Fig. S2

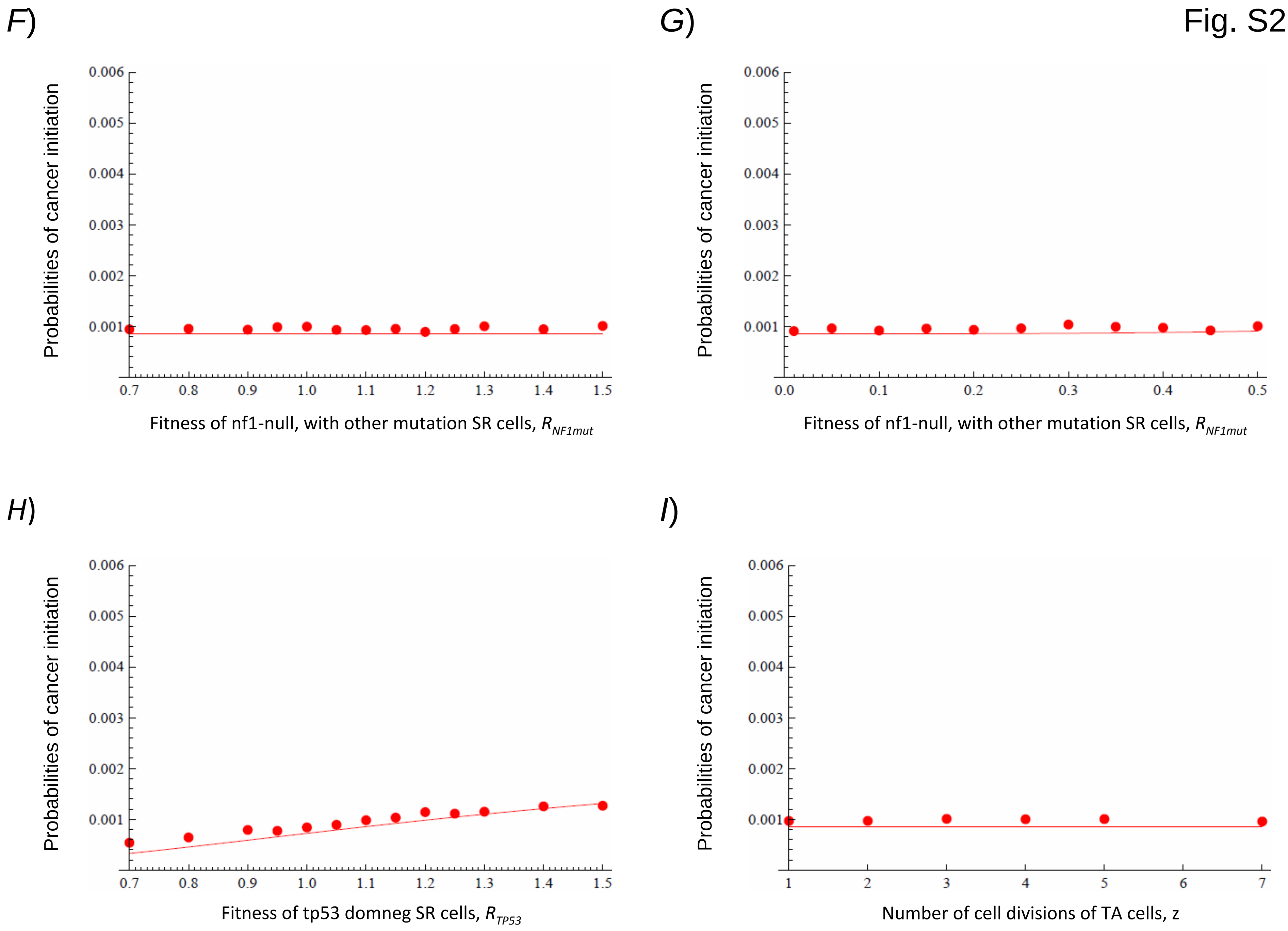

F)
Probabilities of cancer initiation
0.006
0.005
0.004
0.003
0.002
0.001
0.7 0.8 0.9 1.0 1.1 1.2 1.3 1.4 1.5
Fitness of nf1-null, with other mutation SR cells, R_{NF1mut}
G)
Probabilities of cancer initiation
0.006
0.005
0.004
0.003
0.002
0.001
0.0 0.1 0.2 0.3 0.4 0.5
Fitness of nf1-null, with other mutation SR cells, R_{NF1mut}
H)
Probabilities of cancer initiation
0.006
0.005
0.004
0.003
0.002
0.001
0.7 0.8 0.9 1.0 1.1 1.2 1.3 1.4 1.5
Fitness of tp53 domneg SR cells, R_{TP53}
I)
Probabilities of cancer initiation
0.006
0.005
0.004
0.003
0.002
0.001
1 2 3 4 5 6 7
Number of cell divisions of TA cells, z

Fig. S3

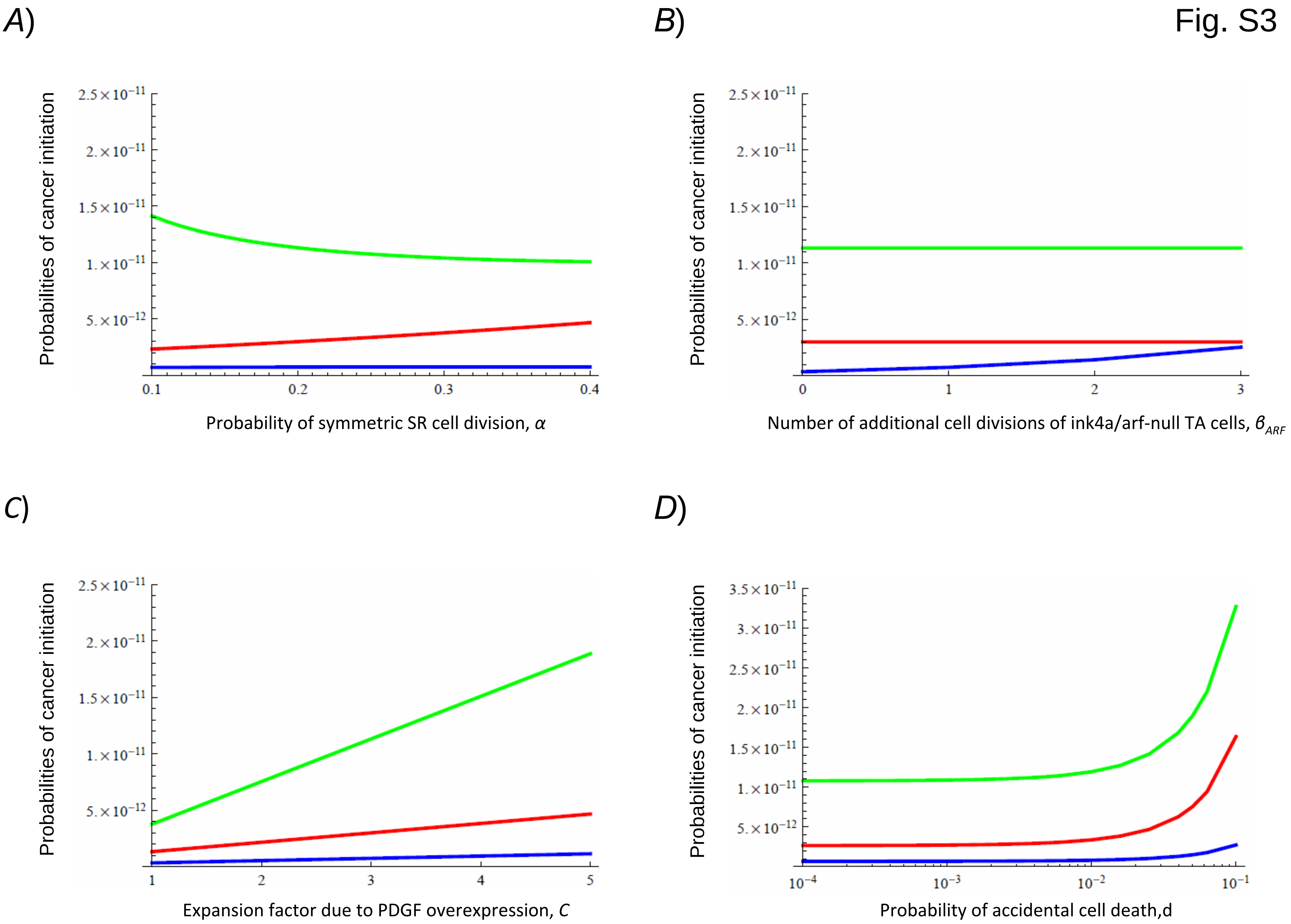

A)
Probabilities of cancer initiation
2.5 × 10⁻¹¹
2. × 10⁻¹¹
1.5 × 10⁻¹¹
1. × 10⁻¹¹
5. × 10⁻¹²
0.1
0.2
0.3
0.4
Probability of symmetric SR cell division, α
B)
Probabilities of cancer initiation
2.5 × 10⁻¹¹
2. × 10⁻¹¹
1.5 × 10⁻¹¹
1. × 10⁻¹¹
5. × 10⁻¹²
0
1
2
3
Number of additional cell divisions of ink4a/arf-null TA cells, β_{ARF}
C)
Probabilities of cancer initiation
2.5 × 10⁻¹¹
2. × 10⁻¹¹
1.5 × 10⁻¹¹
1. × 10⁻¹¹
5. × 10⁻¹²
1
2
3
4
5
Expansion factor due to PDGF overexpression, C
D)
Probabilities of cancer initiation
3.5 × 10⁻¹¹
3. × 10⁻¹¹
2.5 × 10⁻¹¹
2. × 10⁻¹¹
1.5 × 10⁻¹¹
1. × 10⁻¹¹
5. × 10⁻¹²
10⁻⁴
10⁻³
10⁻²
10⁻¹
Probability of accidental cell death,d

Fig. S3

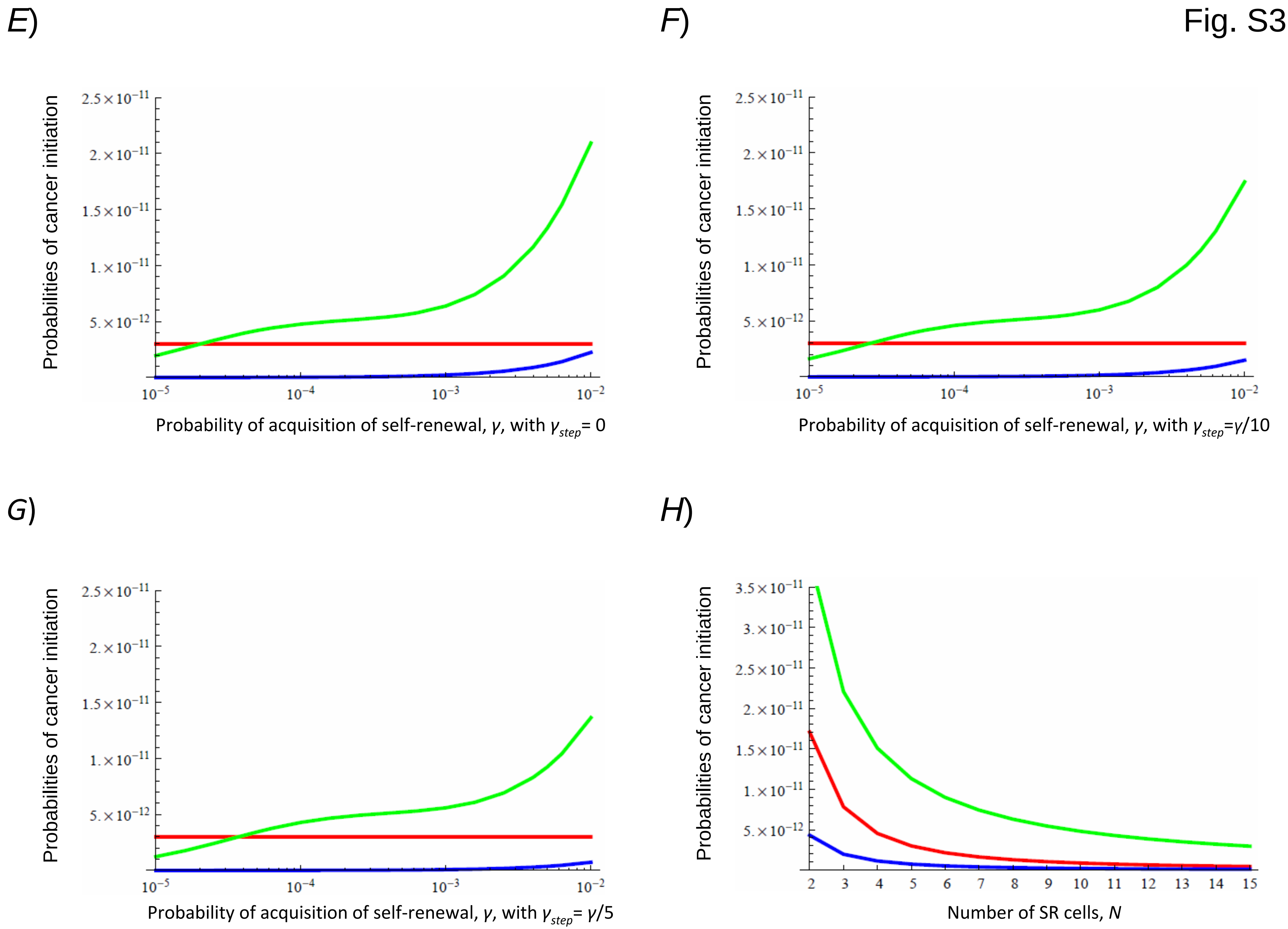
E)
Probabilities of cancer initiation
2.5×10⁻¹¹
2.×10⁻¹¹
1.5×10⁻¹¹
1.×10⁻¹¹
5.×10⁻¹²
10⁻⁵
10⁻⁴
10⁻³
10⁻²
Probability of acquisition of self-renewal, γ, with γstep= 0
F)
Probabilities of cancer initiation
2.5×10⁻¹¹
2.×10⁻¹¹
1.5×10⁻¹¹
1.×10⁻¹¹
5.×10⁻¹²
10⁻⁵
10⁻⁴
10⁻³
10⁻²
Probability of acquisition of self-renewal, γ, with γstep=γ/10
G)
Probabilities of cancer initiation
2.5×10⁻¹¹
2.×10⁻¹¹
1.5×10⁻¹¹
1.×10⁻¹¹
5.×10⁻¹²
10⁻⁵
10⁻⁴
10⁻³
10⁻²
Probability of acquisition of self-renewal, γ, with γstep= γ/5
H)
Probabilities of cancer initiation
3.5×10⁻¹¹
3.×10⁻¹¹
2.5×10⁻¹¹
2.×10⁻¹¹
1.5×10⁻¹¹
1.×10⁻¹¹
5.×10⁻¹²
2 3 4 5 6 7 8 9 10 11 12 13 14 15
Number of SR cells, N

Fig. S3

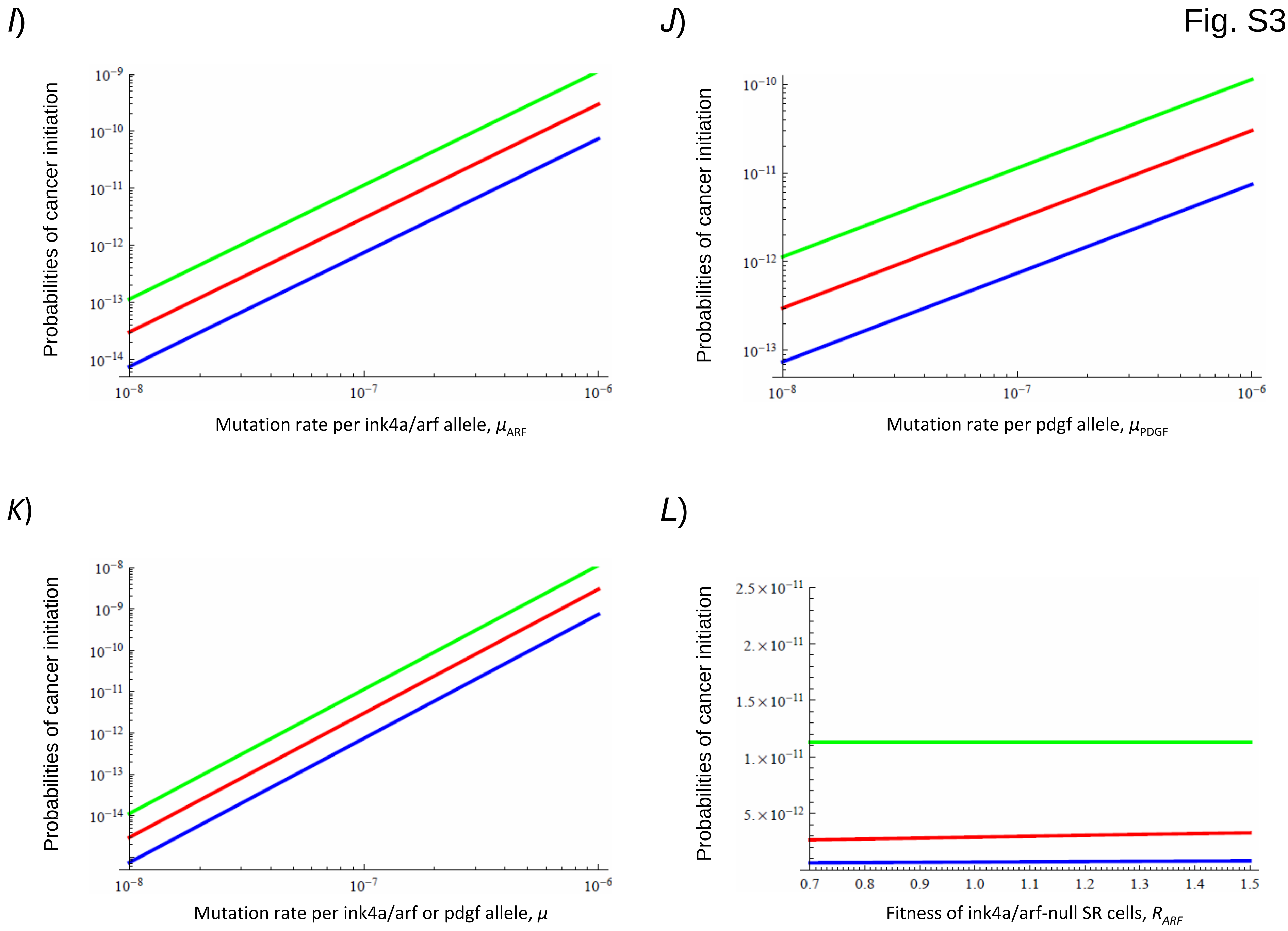

I)
Probabilities of cancer initiation
10^−9
10^−10
10^−11
10^−12
10^−13
10^−14
10^−8
10^−7
10^−6
Mutation rate per ink4a/arf allele, μ_{ARF}
J)
Probabilities of cancer initiation
10^−10
10^−11
10^−12
10^−13
10^−8
10^−7
10^−6
Mutation rate per pdgf allele, μ_{PDGF}
K)
Probabilities of cancer initiation
10^−8
10^−9
10^−10
10^−11
10^−12
10^−13
10^−14
10^−8
10^−7
10^−6
Mutation rate per ink4a/arf or pdgf allele, μ
L)
Probabilities of cancer initiation
2.5 × 10^−11
2. × 10^−11
1.5 × 10^−11
1. × 10^−11
5. × 10^−12
0.7
0.8
0.9
1.0
1.1
1.2
1.3
1.4
1.5
Fitness of ink4a/arf-null SR cells, R_{ARF}

M)

Fig. S3

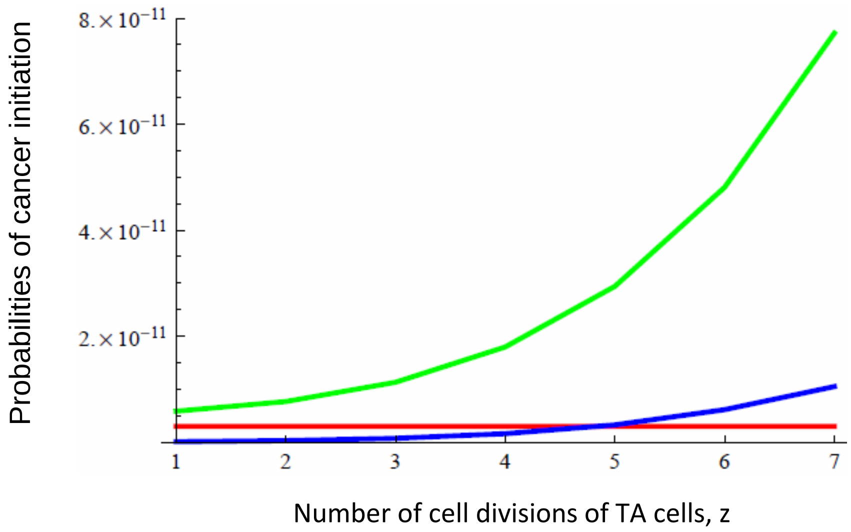

8. × 10⁻¹¹
6. × 10⁻¹¹
4. × 10⁻¹¹
2. × 10⁻¹¹
1
2
3
4
5
6
7
Probabilities of cancer initiation
Number of cell divisions of TA cells, z

Fig. S4

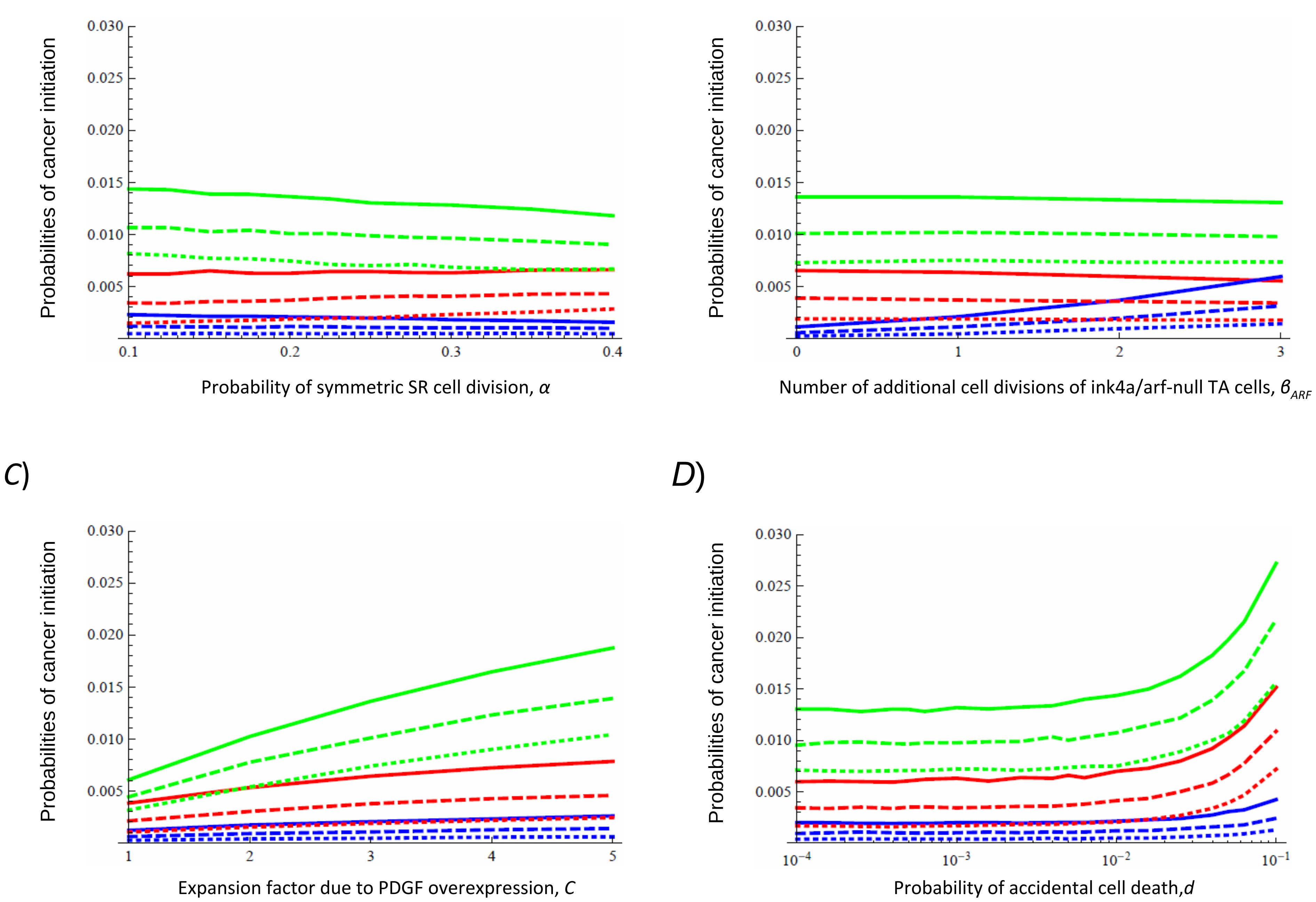
A)
Probabilities of cancer initiation
0.030
0.025
0.020
0.015
0.010
0.005
0.1
0.2
0.3
0.4
Probability of symmetric SR cell division, α
B)
Probabilities of cancer initiation
0.030
0.025
0.020
0.015
0.010
0.005
0
1
2
3
Number of additional cell divisions of ink4a/arf-null TA cells, β_{ARF}
C)
Probabilities of cancer initiation
0.030
0.025
0.020
0.015
0.010
0.005
1
2
3
4
5
Expansion factor due to PDGF overexpression, C
D)
Probabilities of cancer initiation
0.030
0.025
0.020
0.015
0.010
0.005
10^{-4}
10^{-3}
10^{-2}
10^{-1}
Probability of accidental cell death, d

Fig. S4

E)

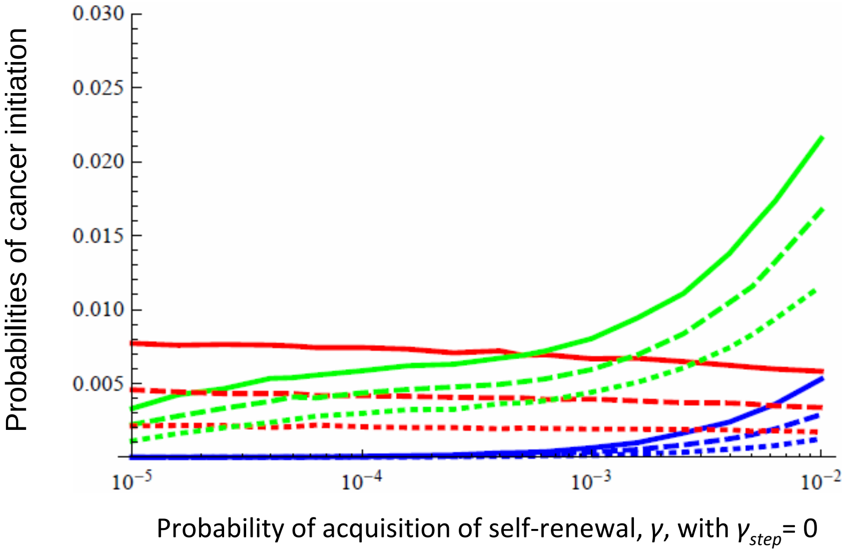

Probabilities of cancer initiation
0.030
0.025
0.020
0.015
0.010
0.005
10^{-5}
10^{-4}
10^{-3}
10^{-2}
Probability of acquisition of self-renewal, γ, with γ_{step}= 0

F)

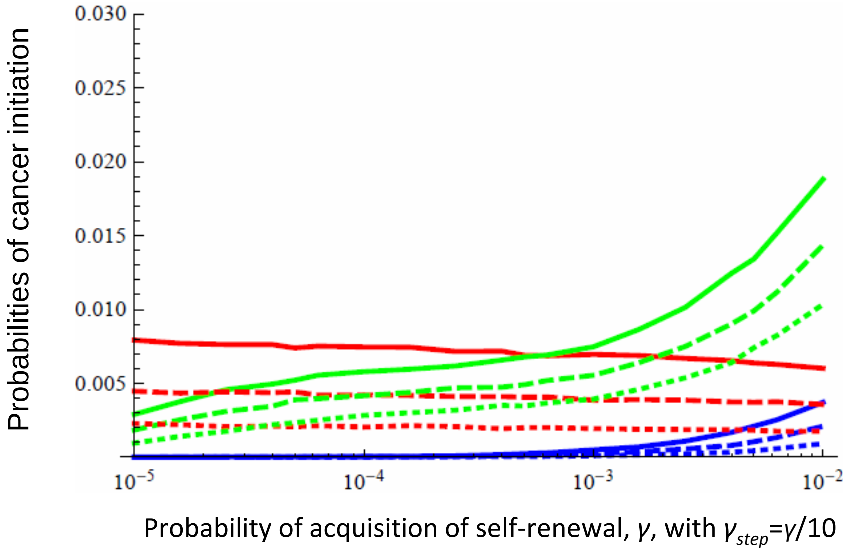

Probabilities of cancer initiation
0.030
0.025
0.020
0.015
0.010
0.005
10^{-5}
10^{-4}
10^{-3}
10^{-2}
Probability of acquisition of self-renewal, γ, with γ_{step}=γ/10

G)

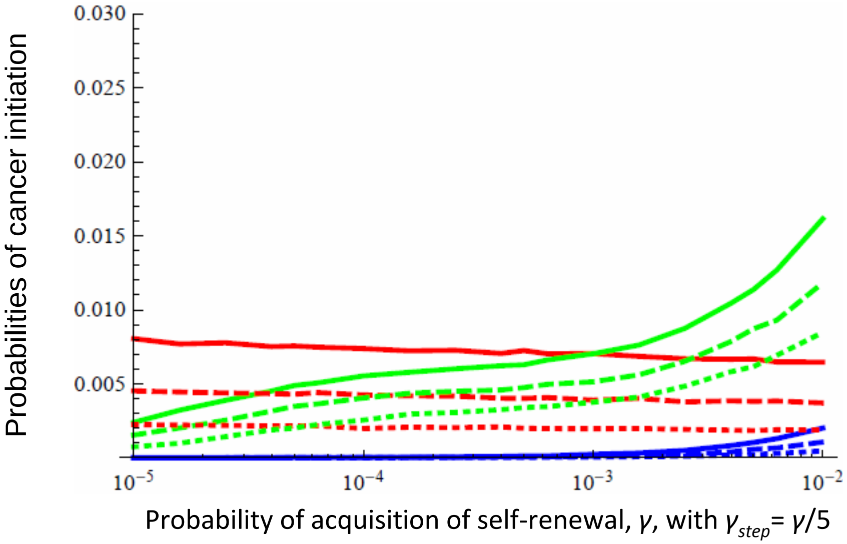

Probabilities of cancer initiation
0.030
0.025
0.020
0.015
0.010
0.005
10^{-5}
10^{-4}
10^{-3}
10^{-2}
Probability of acquisition of self-renewal, γ, with γ_{step}= γ/5

H)

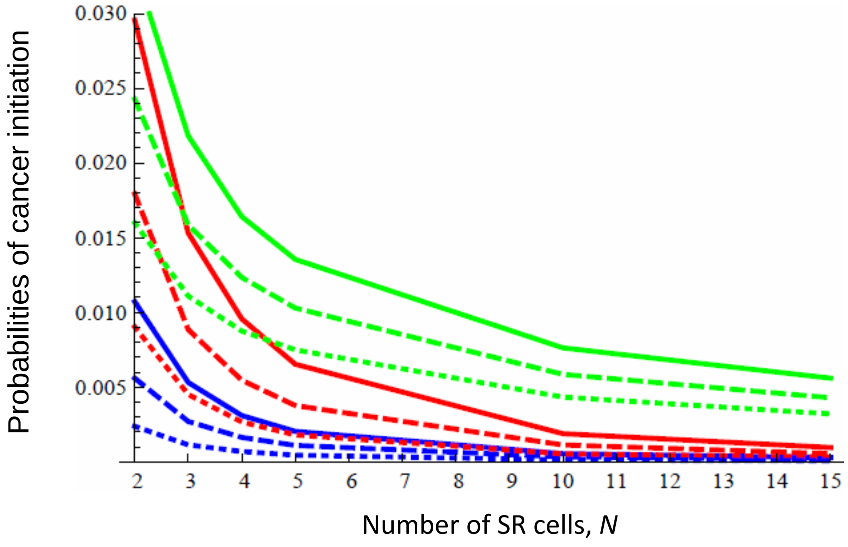

Probabilities of cancer initiation
0.030
0.025
0.020
0.015
0.010
0.005
2
3
4
5
6
7
8
9
10
11
12
13
14
15
Number of SR cells, *N*

Fig. S4

I)

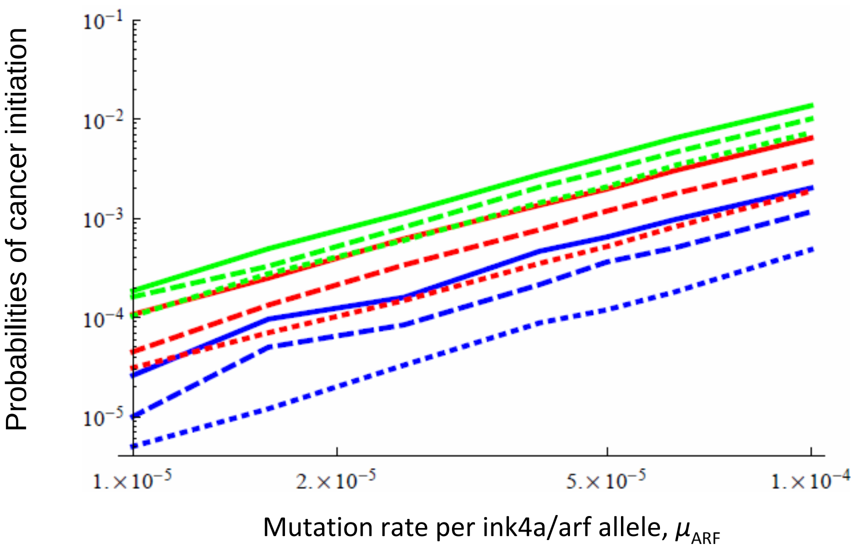
Probabilities of cancer initiation
10^{-1}
10^{-2}
10^{-3}
10^{-4}
10^{-5}
$1.\times10^{-5}$
$2.\times10^{-5}$
$5.\times10^{-5}$
$1.\times10^{-4}$
Mutation rate per ink4a/arf allele, μ_{ARF}

J)

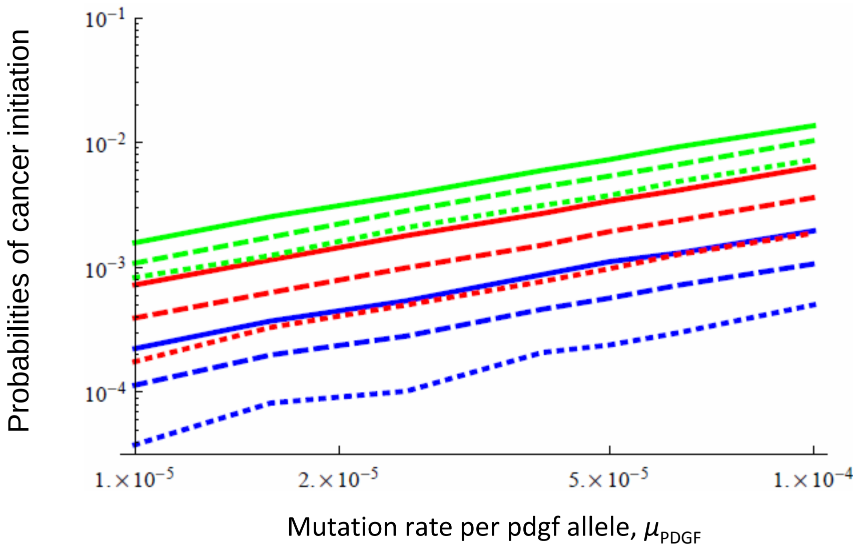
Probabilities of cancer initiation
10^{-1}
10^{-2}
10^{-3}
10^{-4}
$1.\times10^{-5}$
$2.\times10^{-5}$
$5.\times10^{-5}$
$1.\times10^{-4}$
Mutation rate per pdgf allele, μ_{PDGF}

K)

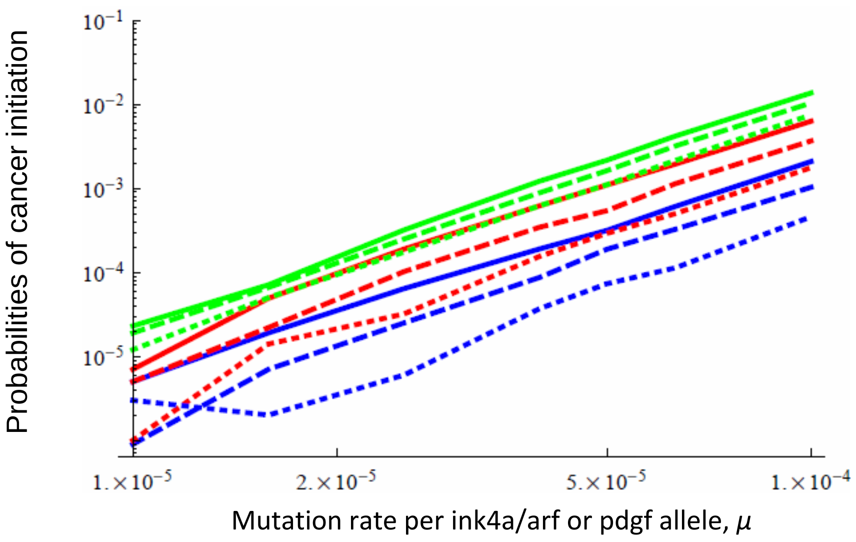
Probabilities of cancer initiation
10^{-1}
10^{-2}
10^{-3}
10^{-4}
10^{-5}
$1.\times10^{-5}$
$2.\times10^{-5}$
$5.\times10^{-5}$
$1.\times10^{-4}$
Mutation rate per ink4a/arf or pdgf allele, μ

L)

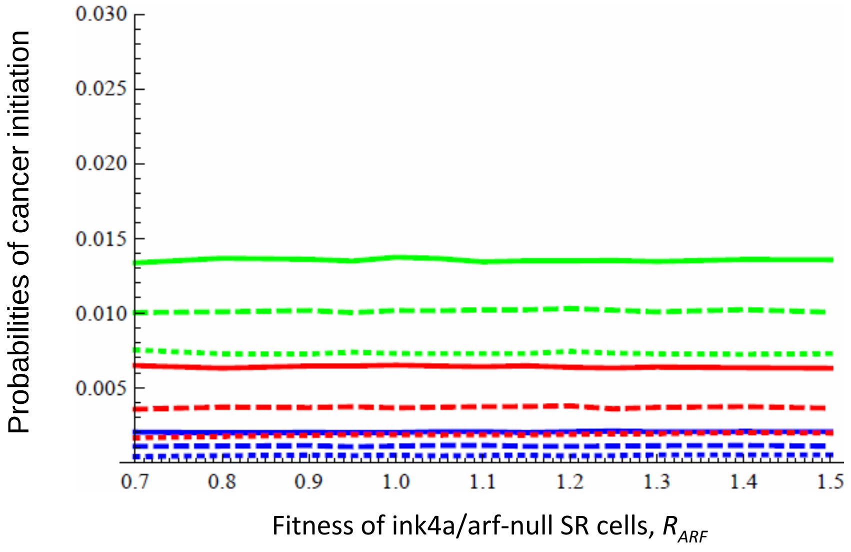
Probabilities of cancer initiation
0.030
0.025
0.020
0.015
0.010
0.005
0.7
0.8
0.9
1.0
1.1
1.2
1.3
1.4
1.5
Fitness of ink4a/arf-null SR cells, R_{ARF}

M)

Fig. S4

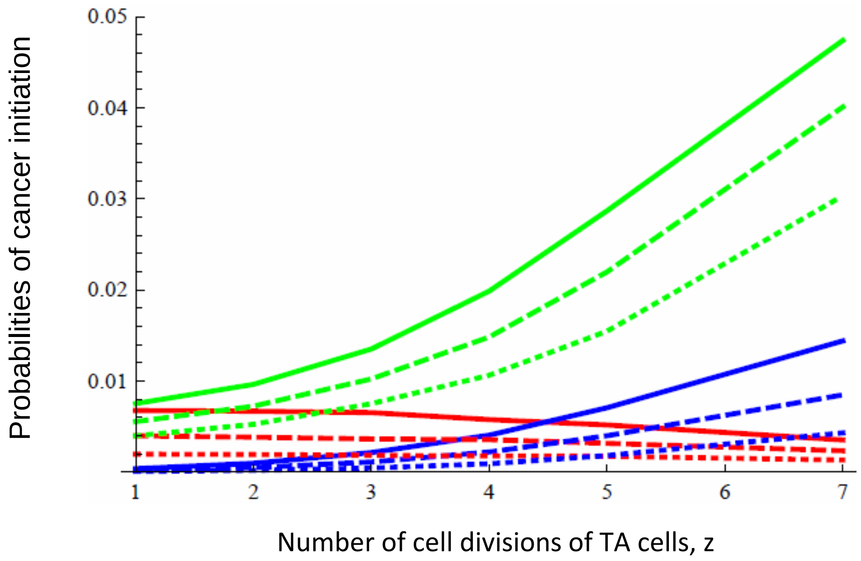
0.05
0.04
0.03
0.02
0.01
Probabilities of cancer initiation
1
2
3
4
5
6
7
Number of cell divisions of TA cells, z

Fig. S5

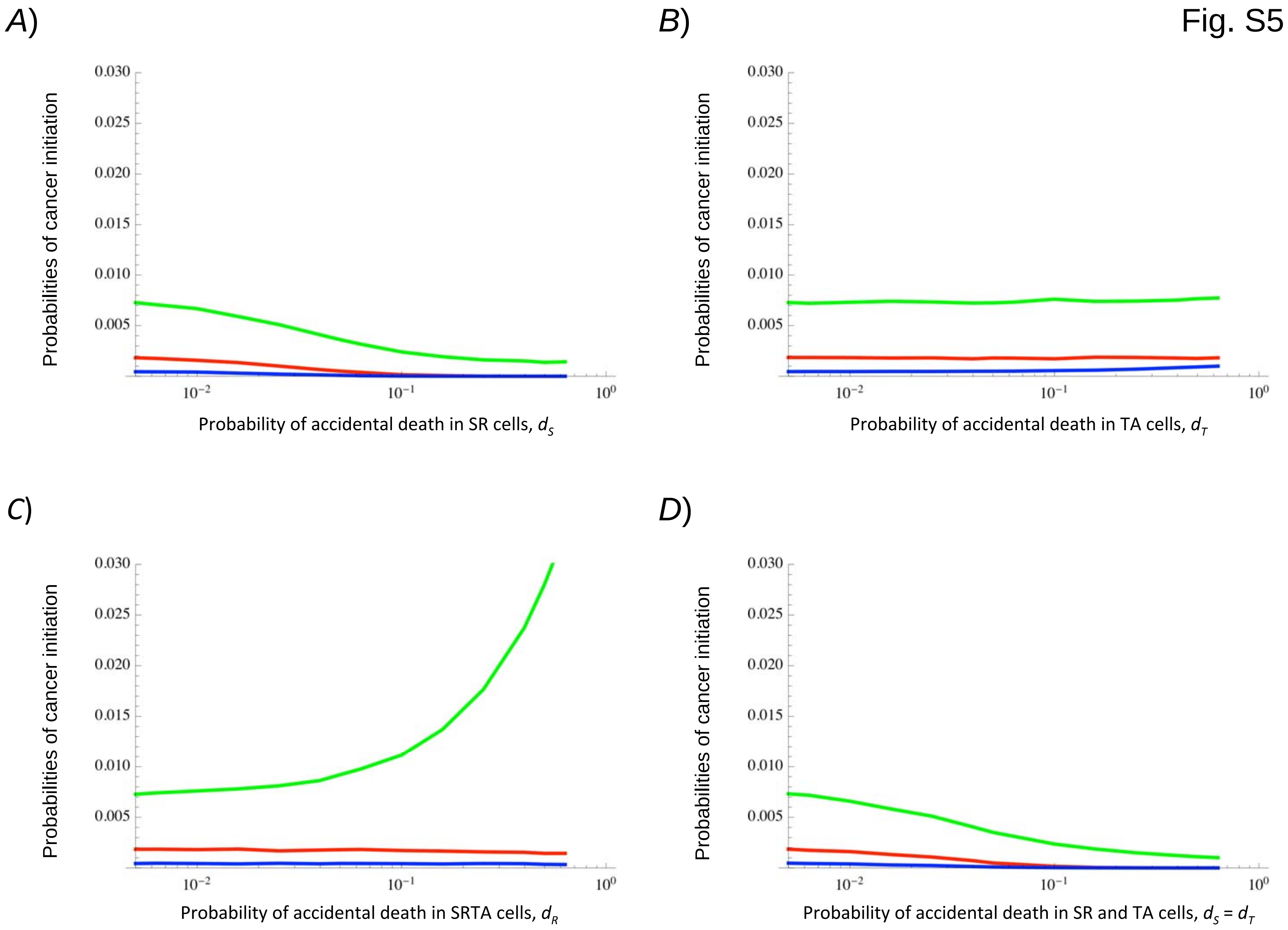

A)
0.030
0.025
0.020
0.015
0.010
0.005
Probabilities of cancer initiation
10^{-2}
10^{-1}
10^{0}
Probability of accidental death in SR cells, d_S
B)
0.030
0.025
0.020
0.015
0.010
0.005
Probabilities of cancer initiation
10^{-2}
10^{-1}
10^{0}
Probability of accidental death in TA cells, d_T
C)
0.030
0.025
0.020
0.015
0.010
0.005
Probabilities of cancer initiation
10^{-2}
10^{-1}
10^{0}
Probability of accidental death in SRTA cells, d_R
D)
0.030
0.025
0.020
0.015
0.010
0.005
Probabilities of cancer initiation
10^{-2}
10^{-1}
10^{0}
Probability of accidental death in SR and TA cells, $d_S = d_T$

Fig. S5

E)

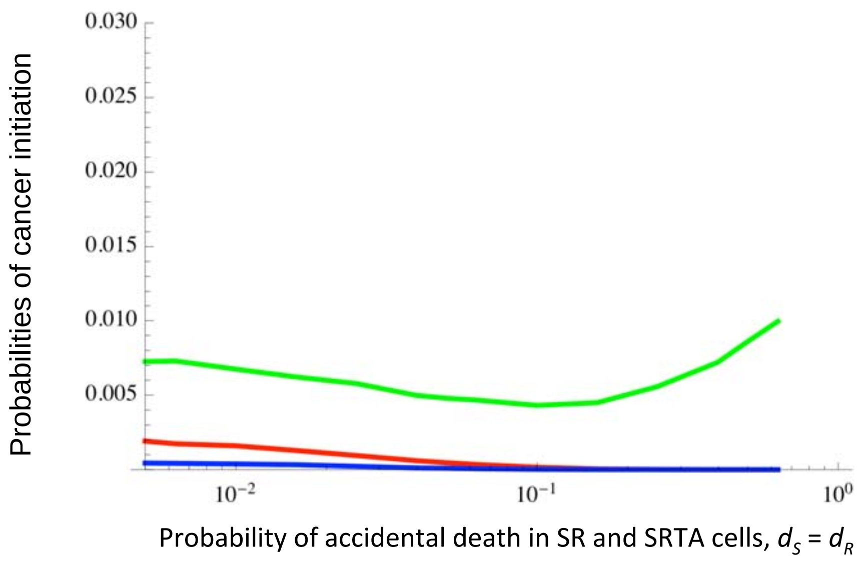
0.030
0.025
0.020
0.015
0.010
0.005
Probabilities of cancer initiation
10^{-2}
10^{-1}
10^{0}
Probability of accidental death in SR and SRTA cells, $d_S = d_R$

F)

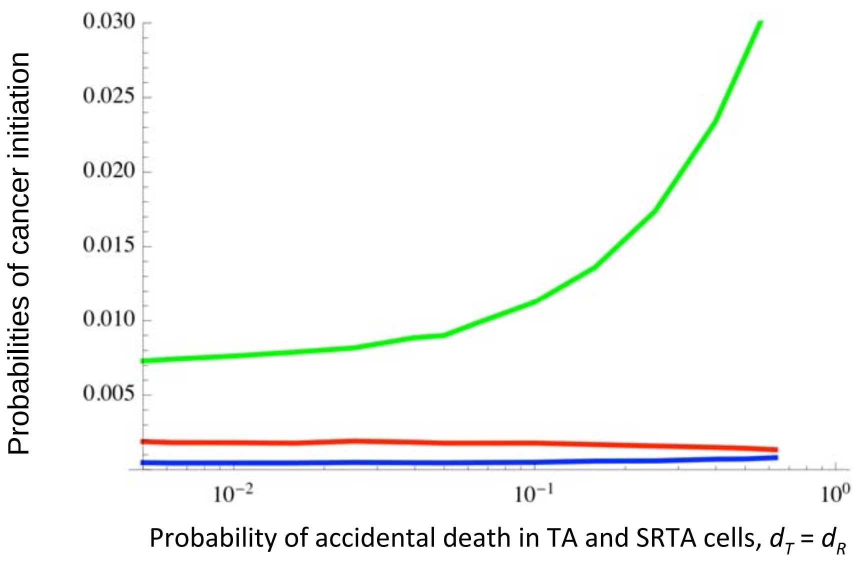
0.030
0.025
0.020
0.015
0.010
0.005
Probabilities of cancer initiation
10^{-2}
10^{-1}
10^{0}
Probability of accidental death in TA and SRTA cells, $d_T = d_R$
